# Cell-penetrating peptide sequence and modification dependent uptake and subcellular distribution of green florescent protein in different cell lines

**DOI:** 10.1038/s41598-019-42456-8

**Published:** 2019-04-18

**Authors:** Sanjay G. Patel, Edward J. Sayers, Lin He, Rohan Narayan, Thomas L. Williams, Emily M. Mills, Rudolf K. Allemann, Louis Y. P. Luk, Arwyn T. Jones, Yu-Hsuan Tsai

**Affiliations:** 1 School of Chemistry, Cardiff University, Cardiff, CF10 3AT UK; 2 School of Pharmacy and Pharmaceutical Sciences, Cardiff University, Cardiff, CF10 3NB UK

**Keywords:** Protein delivery, Chemical tools

## Abstract

Protein therapy holds great promise for treating a variety of diseases. To act on intracellular targets, therapeutic proteins must cross the plasma membrane. This has previously been achieved by covalent attachment to a variety of cell-penetrating peptides (CPPs). However, there is limited information on the relative performance of CPPs in delivering proteins to cells, specifically the cytosol and other intracellular locations. Here we use green fluorescent protein (GFP) as a model cargo to compare delivery capacity of five CPP sequences (Penetratin, R8, TAT, Transportan, Xentry) and cyclic derivatives in different human cell lines (HeLa, HEK, 10T1/2, HepG2) representing different tissues. Confocal microscopy analysis indicates that most fusion proteins when incubated with cells at 10 µM localise to endosomes. Quantification of cellular uptake by flow cytometry reveals that uptake depends on both cell type (10T1/2 > HepG2 > HeLa > HEK), and CPP sequence (Transportan > R8 > Penetratin≈TAT > Xentry). CPP sequence cyclisation or addition of a HA-sequence increased cellular uptake, but fluorescence was still contained in vesicles with no evidence of endosomal escape. Our results provide a guide to select CPP for endosomal/lysosomal delivery and a basis for developing more efficient CPPs in the future.

## Introduction

The delivery of functional proteins to cells offers potential for therapeutic intervention. Many human diseases are associated with malfunction or dysregulation of a specific protein, and provision of an intact protein to the diseased cell is a viable alternative to peptide and nucleotide based therapies^1^. Small-molecule drugs cannot normally mimic the highly specific and complex roles of proteins in cells and often generate adverse side effects. Protein therapeutics are generally safer than genetic approaches as they do not require genome modifications, which can lead to the silencing of indispensable genes or induction of tumourigenesis^2^. Developing protein therapeutics is also financially appealing, as the average clinical development and approval time is shorter than for small-molecule drugs^3^ and wide-reaching patent protection can often be obtained^1^.

Unlike conventional small-molecule drugs, most proteins are large and hydrophilic. Such characteristics mean they do not pass directly through the plasma membrane which restricts their use as therapeutics. Cell-penetrating peptides (CPPs) have shown potential for the delivery of a wide range of molecules, including large active proteins to enter cells via endocytosis^4–6^. Typically composed of 5–30 amino acids, CPPs are mostly positively charged at physiological pH due to the presence of several arginine and/or lysine residues. Different internalisation mechanisms have been reported to be utilised by CPPs, including direct penetration across the plasma membrane and endosomal uptake via one or several endocytic pathways^7^. Direct translocation is often observed at relatively high CPP concentrations when they are attached to small molecules such as fluorophores^4^. Endocytosis is the most common uptake route for larger cargos such as proteins; hence endosomal escape is required for protein to reach the cytosol and other subcellular locations^8^. Cyclic CPPs have recently been shown to promote highly efficient cytosolic delivery of proteins and RNA^9,10^ suggesting that modifications in sequence arrangement, beyond changing amino acid residues, can enhance delivery capacity.

Since the discovery that HIV Tat protein can penetrate mammalian cells and enter the nucleus^11^, hundreds of peptides that have the capacity to enter cells have been identified and, to various extents, characterised^4^. However, a thorough comparative investigation into the relative potential of different CPPs for intracellular delivery of protein cargos is missing^12–14^. The wealth of available CPPs literature is characterised by significant experimental differences (*e.g*. cargo, CPP concentration, cell type, incubation time, *etc*) thus, it is difficult to draw clear conclusions about the relative performance of different CPPs from these investigations.

Here we compare the ability of five CPPs (Penetratin, R8, TAT, Transportan, Xentry) and their derivatives (cyclic R8, cyclic TAT, HA-TAT) under identical experimental conditions to promote the cellular uptake of green fluorescent protein (GFP) in four cell lines of different tissue origins (HeLa – human cervical epithelial, HEK – human kidney epithelial, 10T1/2 – mouse embryonic, HepG2 – human liver epithelial). GFP was used as the model cargo due to the ease of visualising and quantifying its cellular uptake by fluorescence measurements. The results presented here indicate a very large variation in efficiency of cellular uptake as a function of CPP sequence, cyclisation and cell line. Furthermore, there are also distinct differences in the special subcellular profiles of the fluorescent cargo. The analysis provides a helpful guide for the choice of CPP for cargo delivery into endosomes and lysosomes, and a basis for developing more efficient CPPs in future.

## Results

### Optimising recombinant production of chimeric GFP and CPP fusion protein

In this study, R10, composing of ten consecutive arginine residues, was initially used as the model CPP to establish a recombinant protein expression system. Oligoarginine sequences between eight and twelve consecutive residues long have been shown to transduce small molecules into mammalian cells^14–16^. A DNA sequence corresponding to R10 was added to the N-terminus of superfolder GFP (sfGFP) within the plasmid vector pBAD^17^, which contains an arabinose-inducible *sfGFP* gene with a C-terminal His tag. However, gene expression in *Escherichia coli* TOP10 cells yielded no recombinant R10-sfGFP. In contrast, over 30 mg/L of unmodified sfGFP was obtained under the same expression conditions. It is known that addition of a CPP sequence can have a negative influence on recombinant protein yield^10^, therefore we swapped the position of CPP and His tag (*i.e*. having His tag at the N-terminus and CPP at the C-terminus) in the pBAD plasmid (Fig. S1a) and attempted to produce sfGFP-R10 (Fig. S1b) in TOP10 cells. Although the fluorescent protein obtained after purification showed a single band on SDS-PAGE, mass spectrometry (MS) analysis revealed only two Arg residues present at the C-terminus (Fig. S1c). Such truncations were also observed in sfGFP-TAT and sfGFP-Transportan constructs (Figs. S2–S5). Specifically, only a truncated protein was observed for sfGFP-TAT (Fig. S3), whereas several truncated species and a trace amount of full-length protein were observed for sfGFP-Transportan (Fig. S4). For all experiments, protease inhibitors were present at purification steps and the presence of truncated proteins is most likely due to the endogenous protease activity in *E. coli* TOP10 cells that degrade the proteins in the cytoplasm.

**Figure 1:**
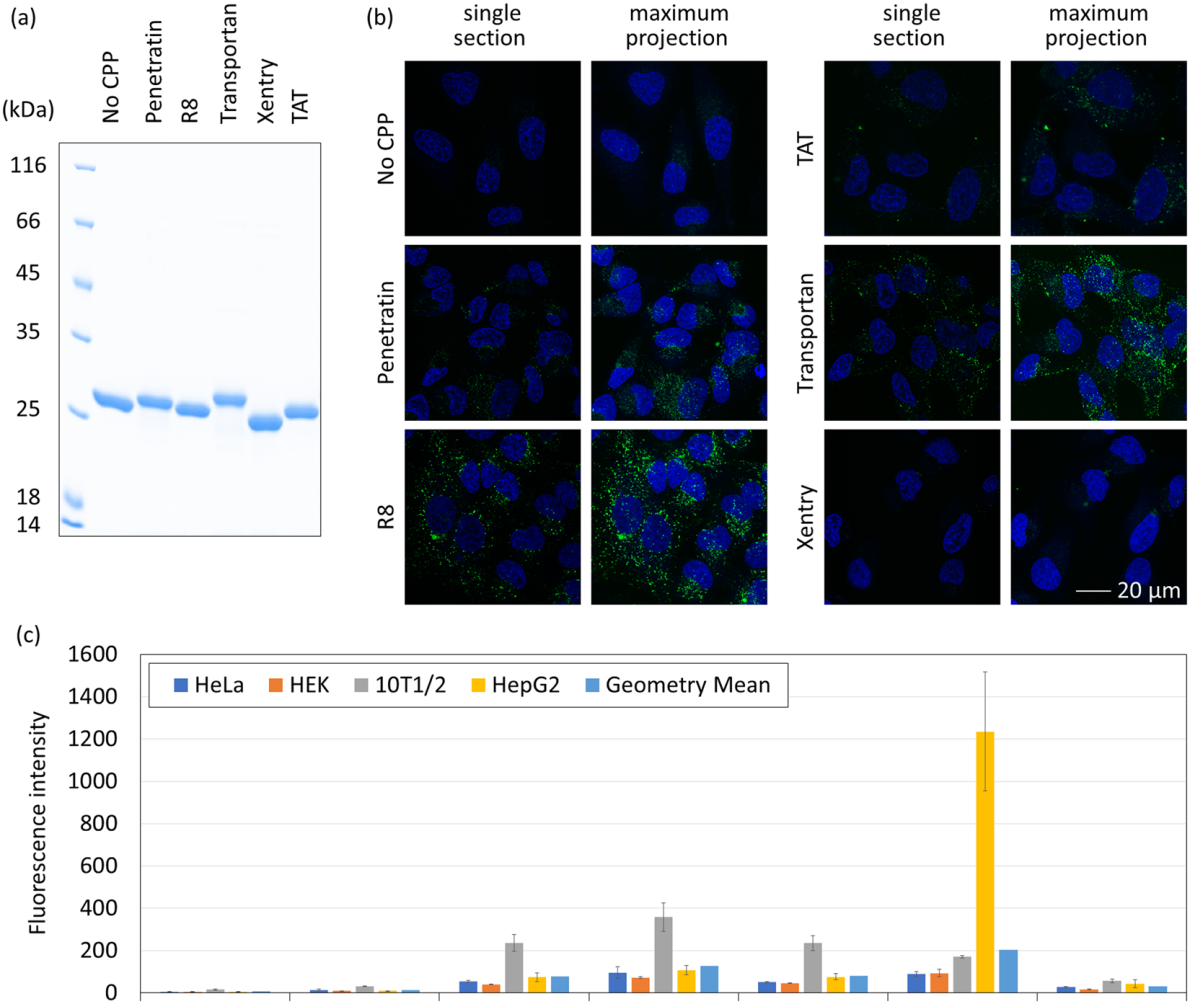
Cellular entry of eGFP-CPP fusion proteins. (**a**) SDS-PAGE of recombinant eGFP-CPP fusion proteins stained using Coomassie blue. (**b**) Confocal microscopy images of HeLa cells treated with eGFP-CPP fusion proteins. Cells were incubated with 10 µM of eGFP-CPP (green) at 37 °C for 1 h before imaging. Nuclei were stained with Hoechst (blue) before imaging. (**c**) Quantification of eGFP-CPP fusion protein uptake by cells. Cells were treated with 10 µM of eGFP-CPP fusion proteins at 37 °C for 1 h before analysis by flow cytometry. Geometric mean values of green fluorescence from living cells were recorded in each experiment. Values shown here are mean ± standard deviation from three independent experiments. There is a statistically significant difference between different CPPs in the same cell line as determined by one-way ANOVA (HeLa: F(6,14) = 17.89, p = 7.9 × 10^−6^; HEK: F(6,14) = 46.48, p = 1.9 × 10^−8^; 10T1/2: F(6,14) = 30.37, p = 3.0 × 10^−7^; HepG2: F(6,14) = 31.42, p = 2.4 × 10^−7^). Exact fluorescence intensity values are shown in Supplementary Table S1. Pairwise statistical analyses are shown in Supplementary Tables S2 and S3.

To minimise the undesired proteolytic cleavage during protein expression, *E. coli* BL21(DE3)pLysS, which lacks the proteases Lon and OmpT, was investigated. Expression using pBAD vectors in BL21(DE3)pLysS gave a lower amount of sfGFP without a CPP present. Therefore, we switched to pEV vector, as this system is proven to produce high levels of enhanced GFP (eGFP) carrying a C-terminal R8 sequence in BL21(DE3)pLysS^18^. Expression of eGFP-R8 in BL21(DE3)pLysS gave a yield similar to that of eGFP without a CPP, and only the full length protein was obtained (Fig. 1a and Table 1). Thus, we decided to switch to R8 and eGFP as the model CPP and the model cargo, respectively. Different CPP sequences were cloned into the C-terminus of GFP in pEV eGFP plasmid (Fig. S6) that contains an inducible *eGFP* gene with an N-terminal His tag. Fusion proteins containing Penetratin, Transportan, Xentry, or TAT were obtained as full-length proteins (Figs. 1 and S7). However, attempts to produce eGFP containing another two commonly used CPPs, Integrin and MAP^14^, were not successful due to a mixture of full-length and truncated proteins being present after purification (Fig. S8). It was not possible to obtain pure full-length eGFP-Integrin or eGFP-MAP by either size-exclusion or ion-exchange chromatography, and the constructs were omitted from subsequent cellular uptake experiments. Additionally, eGFP-Xentry appeared to exist predominantly as a dimer (Fig. S9), most likely due to formation of an intermolecular disulphide linkage from the cysteine residue in the Xentry sequence.

**Table 1:** Molecular weight and expression yield of eGFP-CPP fusion proteins.

Name^a^	CPP Sequence^b^	Calculated MW (Da)	Observed MW (Da)^c^	Yield (mg/L)
No CPP	—	27875.52	27875.00	32
Penetratin	RQIKIWFQNRRMKWKK	30104.26	30104.00	21
R8	RRRRRRRR	29125.02	29124.50	30
TAT	YGRKKRRQRRR	29417.35	29417.00	31
Transportan	GWTLNSAGYLLGKINLKALAALAKKIL	30698.98	30698.50	11
Xentry	LCLRPVG	28614.47	28615.50	31
cR8	CRRRRRRRRC	29329.30	29329.00	27
cTAT	CYGRKKRRQRRRC	29621.63	29620.00	13
HA-TAT	GDIMGEWGNEIFGAIAGFLGYGRKKRRQRRR	31453.64	31453.50	14

^a^All constructs contain an *N*-terminal His-tag. The exact amino acid sequence of each construct is shown in Supplementary Fig. S6. ^b^R8 sequence is shown in italic and TAT sequence is shown in bold. The two Cys residues in the cR8 and cTAT constructs form a disulphide bond and cyclic structure of CPP. ^c^MS spectra are shown in the Supplementary Fig. S7.

### Cellular uptakes of eGFP-CPP

Confocal microscopy was initially employed to assess internalisation of the different eGFP-CPP fusion proteins. HeLa cells were incubated with 10 µM of proteins at 37 °C for 1 h under cell culture conditions then washed with heparin sulphate solution and culture media before imaging. Heparin sulphate is highly negatively charged and should minimise the presence of positively charged eGFP-CPP fusion proteins that associated with the plasma membrane^4^. In the absence of CPP, no cell-associated fluorescence was observed (Fig. 1b), refuting the possibility that His tag or eGFP itself may facilitate cellular uptake. Prominent eGFP-R8 and eGFP-Transportan uptake was observed in punctate structures but the fluorescence subcellular distribution and thus labelling pattern was very different. While eGFP-R8 is enriched in a perinuclear localisation, eGFP-Transportan displays punctate fluorescence towards the periphery of the cells. Both eGFP-Penetratin and eGFP-TAT also display perinuclear enrichment, similar to that seen using eGFP-R8, albeit with reduced levels of internalisation. With all three of these sequences (R8, Penetratin and TAT) being predominantly cationic, there may be similarities in terms of uptake mechanism over the more hydrophobic eGFP-Transportan. eGFP-Xentry also contains mainly hydrophobic residues, however, due to its low uptake levels it is difficult to determine if there are similarities between this sequence and eGFP-Transportan with respects to subcellular distribution.

The punctate signal seen in all CPP constructs is indicative of endocytosis being involved in the uptake mechanism, the lack of unified green fluorescence signals in the cytosol suggests that none of the CPPs promote high levels of endosomal escape. We cannot however, rule out the possibility that a small fraction of proteins has entered the cytosol but falls under the detection limit of confocal microscopy.

To quantify cellular uptake across a range of differing cell lines, cells treated with eGFP-CPP constructs were subjected to flow cytometry analysis. Following incubation with each eGFP-CPP protein at 37 °C for 1 h, cells were washed with heparin sulphate solution and culture media, trypsinised and subjected to flow cytometry. The mean fluorescent intensity was calculated from three biological replicas of the geometric mean fluorescence of each eGFP-CPP fusion protein in the different cell lines (Fig. 1c and Table S1). While cells treated with eGFP without a CPP showed negligible fluorescence, cells incubated with all the other eGFP-CPP fusion proteins displayed a substantial fluorescence reading (Table S2). The fluorescence intensity depended on both cell type and CPP sequence. 10T1/2 cells showed the highest level of uptake of the cationic peptides (Penetratin, R8 and TAT), and the increased uptake was seen for all peptides when compared to the internalisation by both HeLa and HEK cells (Tables S1 and S3). It is interesting to note the very high uptake of eGFP-Transportan seen in HepG2 cells that is not seen with any of the other peptides that show comparable uptake. Overall, the performance of individual CPPs in the four cell lines has the general trend of Transportan > R8 > Penetratin≈TAT > Xentry (Tables 2 and S2). It is noteworthy that none of the CPPs seem to cause any toxicity in HeLa cells (Fig. S10).

**Table 2:** Relative performances of five CPPs in four cell lines.

	Cell Only	No CPP	Penetratin	R8	TAT	Transportan	Xentry
Mean^a^	7	14	78	127	79	189	31
Std^a^	1.59	1.65	1.95	1.84	1.91	2.61	1.58

^a^Geometric mean and geometric standard deviation are shown here.

### Modification of CPP to enhance cellular uptake

Cyclisation of CPPs have recently been shown to increase cellular uptake^19^ and promote direct translocation of CPP-conjugated proteins into the cytosol^9,10^. To form a cyclic structure, we introduced a cysteine residue at each side of the CPP sequence (*i.e*. Cys-CPP-Cys), and a disulphide bond was expected to form between the two cysteine residues and cyclise two ends of the CPP. This should occur in all tested CPPs apart from Xentry which contains an endogenous cysteine.

In addition to cyclisation, we also investigated if addition of a well characterised endosomal escape sequence from the influenza virus haemagglutinin protein (HA)^20,21^ enhanced cellular uptake and/or cytosolic delivery. The peptide was introduced between eGFP and TAT; TAT-HA has been shown to deliver proteins into cells, specifically the cytosol and nucleus^20,21^.

Following the same expression protocol, we were able to obtain eGFP-HA-TAT and eGFP with a C-terminal cyclic Penetratin (cPenetratin), cyclic R8 (cR8) or cyclic TAT (cTAT) (Fig. S11), although the yield of eGFP-cPenetratin (3.5 mg/L) was significantly lower than the other variants (Table 1). Only truncated products were observed for eGFP-cTransportan and eGFP-HA-cTAT under the previously validated expression condition. When raising the expression temperature from 20 °C to 37 °C, full-length eGFP-cTransportan was discovered in the insoluble fraction upon cell lysis. However, refolding of insoluble protein under various conditions was not successful (data not shown). Thus, we proceeded the investigation with cPenetratin, cR8, cTAT and HA-TAT.

**Figure 2:**
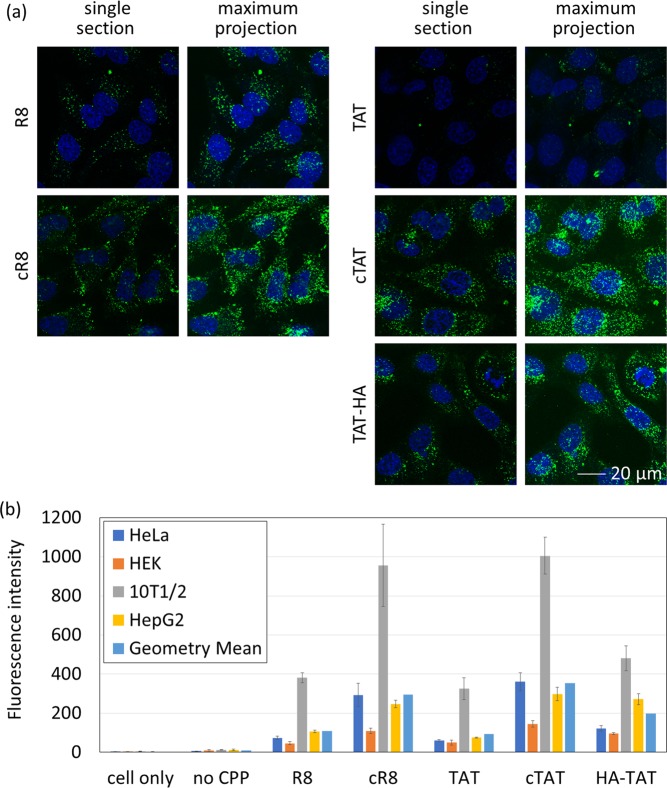
Green fluorescence intensity of cells treated with eGFP proteins fused to a modified CPP. Experimental conditions were the same as described in Fig. 1. (**a**) Confocal microscopy images. (**b**) Quantification by flow cytometry. There is a statistically significant difference between different CPPs in the same cell line as determined by one-way ANOVA (HeLa: F(6,14) = 46.42, p = 2.0 × 10^−6^; HEK: F(6,14) = 115.5, p = 4.0 × 10^−11^; 10T1/2: F(6,14) = 37.13, p = 8.0 × 10^−8^; HepG2: F(6,14) = 136.5, p = 1.0 × 10^−11^). Exact fluorescence intensity values are shown in Supplementary Table S4. Pairwise statistical analyses are shown in Supplementary Tables S5 and S6.

Compared with linear CPPs, confocal microscopy showed a visibly increased fluorescence signal after either cyclisation or addition of the HA peptide (Fig. 2a), a finding that is supported by flow cytometry analysis of the same conjugates (Fig. 2b). Interestingly the localisation of both eGFP-R8 and eGFP-TAT upon cyclisation was different to the linear variants. CPP cyclisation changed the localisation to a more peripheral position within the cell, with a signal more aligned to that seen using eGFP-Transportan. Comparing eGFP-cTAT and eGFP-HA-TAT, both variants also show differences in localisation, with eGFP-cTAT more scattered throughout the cell and eGFP-HA-TAT showing some perinuclear enrichment.

No significant endosomal escape was observed using any of the peptides although, again, low level escape is difficult to detect (Fig. 2a). Attempts to image eGFP-cPenetratin produced images dominated by protein aggregates (Fig. S12). During purification of eGFP-cPenetratin, protein precipitation was frequently observed when concentrating this construct. This was not observed with the other variants, indicating the intrinsic instability of eGFP-cPenetratin.

Flow cytometry analysis in different cell lines confirmed that cyclisation of either R8 or TAT increases cellular uptake (Fig. 2b and Table 3). All cell types showed an increase in uptake in response to cyclisation (Tables S4 and S5) with HeLa producing the largest response with a 4.0- and 5.9-fold increase in fluorescence of R8 and TAT, respectively (*p* = 0.0034 and 0.0004 in t-tests). Addition of the HA sequence to TAT also increased the uptake across all four cell lines. However, this increase was not to the same extent as cTAT. Confocal microscopy of TAT-HA in HeLa cells showed that fluorescence was localised to vesicular structures with no evidence of high level endosomal escape.

**Table 3:** Relative performances of five CPPs in four cell lines.

	Cell Only	No CPP	R8	cR8	TAT	cTAT	HA-TAT
Mean^a^	3	9	112	294	89	356	201
Std^a^	1.31	1.33	2.15	2.12	2.17	1.97	1.91

^a^Geometric mean and geometric standard deviation are shown here.

Since cyclic CPP was reported to enable direct translocation into cells at higher concentration (>50 µM)^9^, HeLa cells were treated with 100 µM eGFP-cR8 or eGFP-cTAT and analysed by confocal microscope (Fig. S13). Cytoplasmic punctate green fluorescence was still observed in all cases, indicating the vast majority of cellular uptake had occurred by endocytosis.

## Discussion

In this work, we directly compared the cell-penetrating ability of seven CPPs, Penetratin, R8, Transportan, Xentry, TAT, TAT-HA, cTAT to deliver eGFP into four different cell lines. The cationic peptides TAT^11^ and Penetratin^22^ were among the first CPPs to be discovered and, along with synthetic oligoarginine sequences, they have been widely used to promote cellular uptake of different cargos *in vitro* and *in vivo*^4^. The more hydrophobic, amphipathic Transportan was found to be the most efficient CPP among 22 sequences for delivering an organic fluorophore into mammalian cells^14^, and our data reveal that Transportan is extremely efficient at delivering eGFP to HepG2 cells. Xentry is a relatively new class of CPP and preferentially enters cells expressing high levels of syndecans (*e.g*. HepG2)^23^. Because of their previously reported ability to deliver small molecule cargoes^14^, we also attempted to assess the capacity of Integrin peptide and MAP^14^ to deliver a protein load, but these constructs proved difficult to express and purify under our optimised conditions.

As many CPPs share common features with anti-microbial peptides^24,25^, we needed to modify our standard bacterial expression system to obtain a yield that would allow for subsequent analysis. Our initial attempt to generate a construct with an N-terminal CPP conjugated to sfGFP in *E. coli* TOP10 produced a low yield of the desired product, despite sfGFP alone being produced at a high yield. The low yield of R10-sfGFP is most likely due to the presence of Arg residues at the N-terminus, which can shorten the half-life of a protein to *ca*. 2 min^26^. To overcome this, CPPs were appended at the C-terminus of the constructs for expression in TOP10 cells. Very low yields were again observed, most likely due to proteolytic cleavage of the recombinant proteins in the cytoplasm. Changing the expression host to BL21(DE3)pLysS, which lacks proteases Lon and OmpT, results in much higher expression. This highlights the importance of using bacteria that have low protease activity in preparing CPP-fused proteins. The decision was also made to switch from sfGFP to eGFP-R8^18^ as this variant is more suitable for production at 37 °C^27^, a temperature we show to reduce truncation artefacts in our system. However, truncated proteins were still observed when producing eGFP-Integrin and eGFP-MAP in BL21(DE3)pLysS (Fig. S8). Integrin and MAP are probably sub-ideal choices of CPP for producing recombinant CPP fusion protein and methods for generating such constructs require optimisation before we can study their delivery capacity.

Addition of a peptide sequence that interacts with endosomal membranes has been shown to influence the ability of CPPs to deliver molecules to the cytosol^8^, a critical step required if the cargo target resides in the cytosol. HA-TAT peptide has previously been used to deliver peptides and proteins into the cytosol and then trafficked to the nucleus^20,21^. Cyclisation of TAT or R8 has been proven to increase the cellular uptake of an organic fluorophore^19^ and was hypothesised that cyclisation increases peptide structural rigidity and contact areas with membrane, leading to enhanced cell penetration^19^. Most importantly, cyclic TAT and cyclic R8 were demonstrated to enable efficient cytosolic delivery of protein cargos through direct plasma membrane translocation^9,10^, a phenomenon not observed in the linear counterparts. Consequently, we put the CPP sequence in-between two cysteine residues, which form a disulphide bond *in situ* and hence generate a cyclic structure. Our strategy allows the production of recombinant proteins containing a cyclic CPP in a single set of procedures. Further, it is economically favoured over previously reported methods where cyclic TAT peptide and the protein cargo were separately produced by solid-phase peptide synthesis and recombinant expression in *E. coli*. They were then subsequently linked together by bioconjugation chemical reactions^9,10^.

Here, cell lines from different species and tissues were tested for cellular uptake of eGFP-CPP. HeLa is a cervical cancer cell line and the most commonly used cell in CPP studies^13^, HEK is a widely-used non-cancer mammalian cell line, 10T1/2 is fibroblast, a different morphology to the other three cell lines which are epithelial, whilst HepG2 is a liver cancer cell line and was used in the previous characterisation of Xentry^23^.

In line with other studies for first line analyses, we have performed these experiments in serum free media to avoid complexation of the constructs to serum proteins such as albumin, while preventing degradation by serum proteases^4,13–16,18–20,23,28–30^. We used confocal microscopy to access the cellular uptake by the fusion of CPP and the subcellular localisation of the model cargo protein, eGFP. Theoretically, positively charged CPP proteins can associate with the negatively charged cell membrane via ionic interaction without being internalised into cells^4^. This possibility was ruled out by the confocal microscopy results, which clearly illustrate that all CPPs can promote the cellular uptake of eGFP, although all eGFP-CPP fusion proteins seem to localise predominantly in endosomes (Fig. 2). Indeed, localisation of protein cargos within the endosomes was expected for all CPPs except HA-TAT, cTAT and cR8 which have been reported to promote release into the cytosol^9,10,20^. It is possible that the HA sequence has a small positive effect on endosomal escape, as the confocal images do not exclude the possibility that a small amount of protein localises in the cytosol or nucleus. For cyclic TAT, it has been reported that this sequence can be internalised into HeLa cells with immediate bioavailability in the cytosol^9^. This conclusion differs from our observations and may be attributed to experimental differences. In the previous report, a mixture of D- and L- amino acids were used and the resulting CPP (rRrGrKkRr) may have alternative biophysical properties^9^. In our studies, the cyclic linkage was made via disulphide bond formation whilst an isopeptide bond between lysine and glutamic acid was used by Nischan *et al*.^9^.

Our quantitative cellular uptake analysis gives a general trend of cTAT > cR8 > HA-TAT≈Transportan > R8 > Penetratin≈TAT > Xentry for the CPPs examined (Tables 2, 3, S2 and S5) and 10T1/2 > HepG2 > HeLa > HEK for the cell lines tested (Tables S1, S3, S4 and S6). Overall, the flow cytometry results are qualitatively in agreement with the confocal microscopy observations (Figs 1 and 2). The observed trend is also in agreement with previous studies using organic fluorophores, where cellular uptake is generally higher in HeLa than HEK cells^14^; Transportan outperformed both Penetratin and TAT^13,14^; and cyclisation increased the cellular uptake promoted by TAT^19^. To our surprise, there was little uptake of eGFP-Xentry in any cell line, including HepG2 which was used to establish this peptide as a CPP for the delivery of organic fluorophores, proteins and nucleic acids^23^. Since replacement of the cysteine residue in the Xentry with a leucine totally abolished the cell-penetrating ability^29^, the minimal uptake of eGFP-Xentry is probably due to the formation of dimer through the cysteine residue in the Xentry sequence. However, cysteine residues are prone to form disulphide bonds under normal atmospheric conditions, so the need of a free cysteine residue for Xentry means that Xentry conjugates have to be preserved in the absence of oxygen and/or with reducing reagents, an unattractive requirement for developing therapeutic proteins. Both HA-TAT and cTAT showed higher cellular uptake as quantified by the fluorescence intensity (Fig. 2b). One possibility for the higher fluorescence intensity observed with eGFP-HA-TAT could be a consequence of the endosomolytic activity of the HA peptide. This peptide has been shown to permeabilise endosomal membranes without releasing the fusion protein cargo to cytosol^28^. Once endosomal membranes are permeabilised, the endosomal pH is likely to increase towards neutral (i.e. cytosolic pH), consequently leading to higher fluorescence intensity of eGFP, which is brighter at pH neutral and basic environments^31^.

Lastly, the effect of cyclisation is in agreement with the previous study using an organic fluorophore^19^, suggesting a universal effectiveness of this approach to enhance cellular uptake.

The localisation of eGFP (after internalisation using different peptides) changes in HeLa cells, and it would be of great interest to deduce the intracellular compartment these peptides are locating to. For treatments of lysosomal storage diseases, rapid trafficking to the lysosome would be of large benefit. However, if a CPP-protein were to be used in conjunction with a method of endosomal escape, slower trafficking to the lysosome may be more appropriate. At 10 µM none of the peptides displayed evidence of cytosolic delivery, although more sensitive methods need to be employed to precisely determine this property. Nevertheless, these CPPs would be useful for delivering protein cargos to endosomes and lysosomes for treatments of diseases where the protein needs, or targets lie in, these organelles^32^.

## Conclusions

Here we provide comparative data for the performance of different CPPs in four cell lines. Our approach is simple, using only an optimised recombinant method. Protease degradation limits the choice of CPPs for obtaining fusion proteins. However, we have shown high yield production of both linear and cyclic TAT and R8 and linear Transportan. Our confocal and flow cytometry data further suggest that cyclisation of CPPs enhances cellular uptake, but it remains to be determined if this modification also increases endosomal escape of protein cargoes (*i.e*. toxins) that have influences on cellular physiology if they reach the cytosol.

## Methods

### Construction of eGFP-CPP plasmids

Plasmids pEV eGFP and pEV eGFP-R8 were described previously^18^. The vector for a plasmid that expresses Penetratin, Xentry, TAT or cTAT fused to eGFP was PCR amplified from pEV eGFP with primers TGAGAATTCAAGCTTAAGCTGAGCAATAACTAGC and CCATGTGGTGGTGGTGGTGGT GCATATGTATATCTC, and the eGFP-CPP sequence was PCR amplified from pEV eGFP with the forward primer CCACCACCACCACCACATGGTGAGCAAGGGCGAG and the corresponding reverse primer AGCTTAAGCTTGAATTCTCAGCCCACGCCCACGCCCGCCAGCGCGCCCAGC GCCAGCACGGTCACCTTGTACAGCTCGTCCATGCCGAGAG (Penetratin), AGCTTAAGCTTGAA TTCTCACGCCAGTTTCAGCGCCGCTTTCAGCGCTTTCAGCGCCAGTTTCAGCGCCAGTTTCTTGTACAGCTCGTCCATGCCGAGAG (Xentry), AGCTTAAGCTTGAATTCTCATTTCTTCCATTTCA TACGGCGGTTCT GGAACCAGATTTTAATTTGACGCTTGTACAGCTCGTCCATGCCGAGAG (TAT) or AGCTTAAGCTTGAATTCTCAGCCCACCGGGCGCAGGCACAGCTTGTACAGCTCGTCCATG CCGAGAG (cTAT). PCR fragments were purified by gel extraction. Gibson assembly of the vector and the corresponding eGFP-CPP fragment afforded plasmids pEV eGFP-CPP. To construct pEV eGFP-Transportan, the vector was obtained from PCR amplification of pEV eGFP with primers AAAATTAACCTGAAAGCGCTGGCGGCGCTGGCGAAAAAAATTCTGTGAGAATTCAAGCTTAAGCTGAGCAATAACTAGC and CCATGTGGTGGTGGTGGTGGTGCATATGTATATCTC, and the insert was PCR amplified from pEV eGFP with primers CCACCACCACCACCACATGG TGAGCAAGGGCGAG and AGCTTAAGCTTGAATTCTCAGCAACGACGACGCTGACGACGTTT TTTACGACCGTAGCACTTGTACAGCTCGTCCATG. PCR fragments were gel purified and subjected to two-piece Gibson assembly to afford pEV eGFP-Transportan. To construct pEV eGFP-TAT-HA, the vector was obtained from PCR amplification of pEV eGFP-TAT with primers GGTTTTCTGGGTTACGGTCGTAAAAAACGTCGTCAGCGTCGTCGTTGAG and CCATGTGGT GGTGGTGGTGGTGCATATGTATATCTC, and the insert was PCR amplified from pEV eGFP-TAT with primers CCACCACCACCACCACATGGTGAGCAAGGGCGAG and AGCTTAAGCTTGAATT CCTACAGCAGGCGGCGCAGGGTGCGTTTGCGCACAATATGGCGGCGGCGCATAAACAGCTTGTACAGCTCGTCCATGCCGAG. PCR fragments were gel purified and subjected to two-piece Gibson assembly to afford pEV eGFP-TAT-HA.

### Expression and purification of recombinant eGFP-CPP fusion proteins

The desired pEV eGFP-CPP plasmid was transformed into *E. coli* BL21(DE3)pLysS and plated on Luria-Bertani (LB) agar containing ampicillin (100 µg/mL) and chloramphenicol (34 µg/mL). A single colony was grown in LB medium (50 mL) containing ampicillin (100 µg/mL) and chloramphenicol (34 µg/mL) at 37 °C with vigorous shaking. After overnight incubation, this culture was diluted 50 times into fresh LB (2 L) containing ampicillin (100 µg/mL) and chloramphenicol (34 µg/mL). The culture was incubated at 37 °C to OD_600_ 0.9 before addition of isopropyl β-D-1-thiogalactopyranoside (IPTG) to a final concentration of 0.5 mM. The culture was further incubated at 20 °C overnight. Cells were then collected by centrifugation at 4 °C and 6,000 × *g* for 20 min. Pellets from the 2 L culture were re-suspended in 40 mL of pre-chilled lysis buffer (pH 7.5, 20 mM Tris-HCl, 400 mM NaCl, 20 mM imidazole) containing protease inhibitor (Roche, #05892953001) and lysozyme (1 mg/mL). The re-suspended cells were sonicated for 10 min (10 sec pulse ON and 30 sec OFF). The cell debris was then removed by centrifugation at 4 °C and 38,000 × *g* for 30 min, and the supernatant (*i.e*. cell lysate) was collected for purification.

XK16/20 column was packed with 10 mL Ni Sepharose 6 Fast Flow resin (GE Healthcare) and incubated with 50 mL of equilibration buffer (pH 7.5, 20 mM Tris-HCl, 400 mM NaCl, 20 mM imidazole), before loading the cell lysate. After the sample passed through the column, the column was washed with wash buffer (pH 6.3, 20 mM Tris-HCl, 400 mM NaCl, 20–50 mM imidazole). eGFP-CPP was eluted with elution buffer (pH 7.5, 20 mM Tris-HCl, 250 mM NaCl, 250 mM imidazole). Fractions were collected and analysed by SDS-PAGE. Fractions containing eGFP-CPP identified by SDS-PAGE were pooled together. Proteins were concentrated to 100 µM and exchanged into phenol red free Dulbecco’s Modified Eagle Medium (DMEM; Life Technologies, #31053-028) using a 10-kDa cut-off membrane (Millipore, #PLGC04310). Protein concentrations were determined by BCA assays.

### Confocal microscopy

HeLa cells were seeded at a density of 3.5 × 10^5^ cells per imaging dish (MatTek, #P35G-1.5-10-C) and grown at 37 °C in a 5% CO_2_ atmosphere in DMEM supplemented with 10% (v/v) FBS for 24 h. Cells were then incubated with 10 μM of the designated eGFP-CPP fusion protein in phenol red free DMEM (Life Technologies, #21063-029) and absence of serum at 37 °C for 1 h, followed by washing three times with heparin sulphate (Sigma, #H3149, 0.5 mg/mL in PBS) and twice with phenol red free DMEM. The nucleus was stained by incubation with Hoechst (1 µg/mL) in phenol red free DMEM at 37 °C for 5 min before imaging on a Leica TCS SP5 confocal microscope.

### Flow cytometry

Cells were seeded at a density of 1 × 10^5^ cells per well in a 24-well plate and grown at 37 °C in a 5% CO_2_ atmosphere in DMEM supplemented with 10% (v/v) FBS for 24 h. Cells were then incubated with 10 μM of the designated eGFP-CPP fusion protein in phenol red free DMEM (Life Technologies, #21063-029) at 37 °C for 1 h, followed by washing three times with heparin sulphate and twice with phenol red free DMEM for two times before addition of trypsin (50 µl/well; Corning, #25-052-CI). Trypsinised cells were diluted with PBS (0.45 mL) and analysed on a Bio-Rad S3e Cell Sorter for minimum 10,000 events per sample. Data were analysed on ProSort software (Bio-Rad), and geometric mean values of green fluorescence (526 nm) of live cells in each sample from excitation with a 488-nm laser were taken for quantification.

## Supplementary information

Supplementary Information

## Data Availability

Information about the data underpinning the results presented here (*i.e*. confocal microscope image and flow cytometry files), including how to access them, can be found in the Cardiff University data catalogue at 10.17035/d.2018.0064167981.
