# Supplementary material for: Cell-penetrating peptide sequence and modification dependent uptake and subcellular distribution of green florescent protein in different cell lines
Source: Sci Rep. 2019 Apr 18;9:6298. doi: 10.1038/s41598-019-42456-8 (PMC6472342; doi:10.1038/s41598-019-42456-8)
Supplement: Supplementary file 1 — Supplementary Information [file 41598_2019_42456_MOESM1_ESM.pdf]

Supplementary Information

Cell-penetrating peptide sequence and modification dependent uptake and subcellular distribution of green florescent protein in different cell lines

Sanjay G. Patel[1], Edward J. Sayers[2], Lin He[2], Rohan Narayan[2], Thomas L. Williams[1], Emily M. Mills[1], Rudolf K. Allemann[1], Louis Y. P. Luk[1], Arwyn T. Jones[2,*] & Yu-Hsuan Tsai[1,*]

[1] School of Chemistry, Cardiff University, Cardiff, CF10 3AT, UK

[2] School of Pharmacy and Pharmaceutical Sciences, Cardiff University, Cardiff CF10 3NB, UK

* Correspondence and requests for materials should be addressed to A.T.J. (email: jonesat@cardiff.ac.uk) or Y.H.T. (email: tsaiy5@cardiff.ac.uk)

Toxicity of eGFP-CPP fusion proteins

HeLa cells were seeded at a density of 2 × 10^4 cells per well in a Corning 96-well plate (Fisher Scientific, #10357891) and grown at 37 °C in a 5% CO_2 atmosphere in DMEM supplemented with 10% (v/v) fetal bovine serum (FBS) for 24 h. Constructs were diluted into DMEM supplemented with 10% FBS to the appropriate concentration, and cells in each well were incubated with 100 µL of the construct solution. After 24 h at 37 °C, 20 µL of CellTiter-Blue (Promega, #G8080) was added to each well. The plate was incubated for another 4 h at 37 °C before analysis on a Perkin Elmer Victor X plate reader (excitation 531 nm; emission 595 nm). Each data point is calculated from three biological replicates (*i.e.* cells split from three different passages), and each biological replicate is calculated from three technical replicates (*i.e.* cells split from the same passage). Values from cell-only (*i.e.* non-treated) wells in each biological replicate were set as 100% viability. Results are shown in Supplementary Figure S10.

(a) DNA sequence of sfGFP-CPP

ATGCACCATCATCATCACCATGTT
AGCAAAGGTGAAGAACTGTTTAC
CGGCGTTGTGCCGATTCTGGTGG
AACTGGATGGTGATGTGAATGGC
CATAAATTTAGCGTTCGTGGCGA
AGGCGAAGGTGATGCGACCAAC
GGTAAACTGACCCTGAAATTTATTTGCACCACCGGTAAACTGCCGGTTCCGTGGCCGACCCTGGTGACCACCCTGACC
TATGGCGTTCAGTGCTTTAGCCGCTATCCGGATCATATGAAACGCCATGATTTCTTTAAAAGCGCGATGCCGGAAGGC
TATGTGCAGGAACGTACCATTAGCTTCAAAGATGATGGCACCTATAAAACCCGTGCGGAAGTTAAATTTGAAGGCGAT
ACCCTGGTGAACCGCATTGAACTGAAAGGTATTGATTTTAAAGAAGATGGCAACATTCTGGGTCATAAACTGGAATAT
AATTTCAACAGCCATAATGTGTATATTACCGCCGATAAACAGAAAAATGGCATCAAAGCGAACTTTAAAATCCGTCAC
AACGTGGAAGATGGTAGCGTGCAGCTGGCGGATCATTATCAGCAGAATACCCCGATTGGTGATGGCCCGGTGCTGCT
GCCGGATAATCATTATCTGAGCACCCAGAGCGTTCTGAGCAAAGATCCGAATGAAAAACGTGATCATATGGTGCTGCT
GGAATTTGTTACCGCCGCGGGCATTACCCACGGTATGGATGAACTGTATAAAGGCAGC[CPP sequence]TAA

**No CPP**	-
**TAT**	CGTCGTCGTCAGCGTCGTAAAAAACGTGGT
**Transportan**	GGCTGGACCCTGAACAGCGCGGGCTATCTGCTGGGCAAA ATTAACCTGAAAGCGCTGGCGGCGCTGGCGAAAAAACTG
**R10**	CGTCGCCGTCGCCGTCGGCGCCGTCGCCGT

(b) Amino acid sequence of sfGFP-CPP

MHHHHHHVSKGEELFTGVVPILVELDGDVNGHKFSVRGE
GEGDATNGKLTLKFICTTGKLPVPWPTLVTTLTYGVQCFSR
YPDHMKRHDFFKSAMPEGYVQERTISFKDDGTYKTRAEVK
FEGDTLVNRIELKGIDFKEDGNILGHKLEYNFNSHNVYITAD
KQKNGIKANFKIRHNVEDGSVQLADHYQQNTPIGDGPVLL
PDNHYLSTQSVLSKDPNEKRDHMVLLEFVTAAGITHGMDELYKGS[CPP sequence]*

**No CPP**	-
**TAT**	RRRQRRKKRG
**Transportan**	GWTLNSAGYLLGKINLKALAALAKKL
**R10**	RRRRRRRRRR

(c)

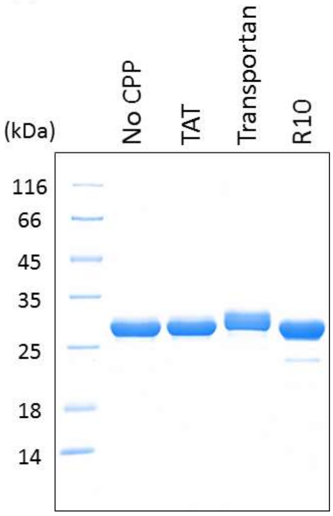

	Theoretical MW (Da)	Observed MW (Da)	Amino Acid Residues Lost in Truncated Proteins
**No CPP**	27827.32	27828.00	
**TAT**	29205.98	-	
	27983.51	27982.00	RRQRRKKRG
**Transportan**	30537.62	30540.00	
	30296.29	30297.00	KL
	29728.56	29729.00	LAALAKKL
	29529.31	29531.00	KALAALAKKL
	29003.66	29004.00	GKINLKALAALAKKL
	28171.69	28173.00	LNSAGYLLGKINLKALAALAKKL
**R10**	29389.20	-	
	28139.70	28137.00	RRRRRRRR

Supplementary Figure S1. sfGFP-CPP fusion proteins expressed from pBAD vectors in *E. coli* TOP10 cells. The proteins contain a His tag on the N-terminus. (a) DNA sequence of sfGFP-CPP. The sequence of His tag is shown in red. (b) Amino acid sequence of sfGFP-CPP. The sequence of His tag is shown in red. (c) SDS-PAGE and observed molecular weight of purified proteins. See Supplementary Figures S2-S5 for the LC chromatograms and MS spectra.

(a) LC chromatogram of sfGFP

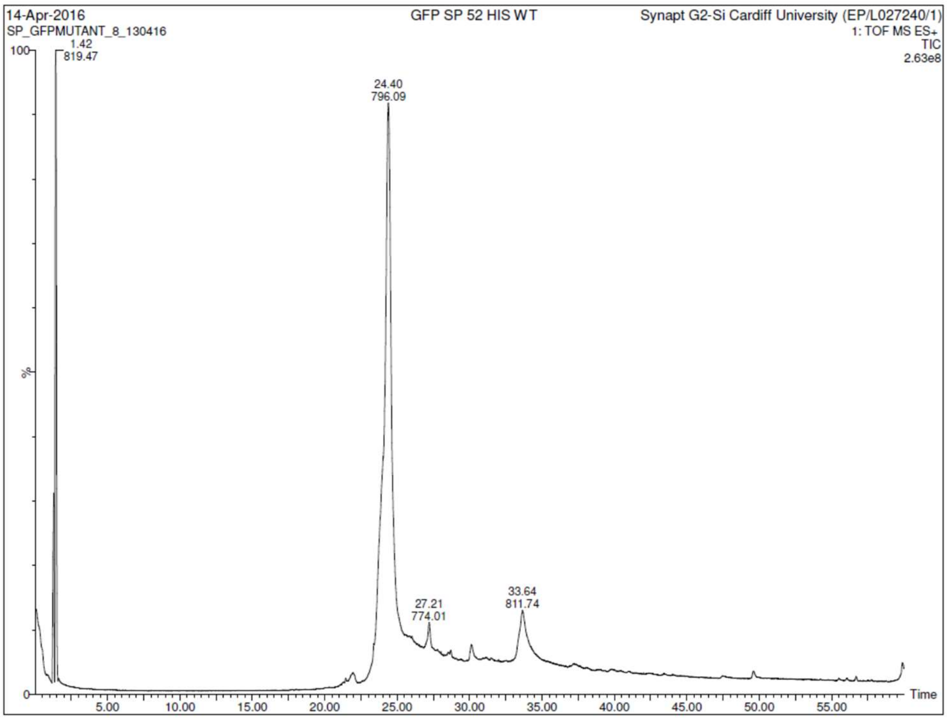

(b) MS of peak at 24.43

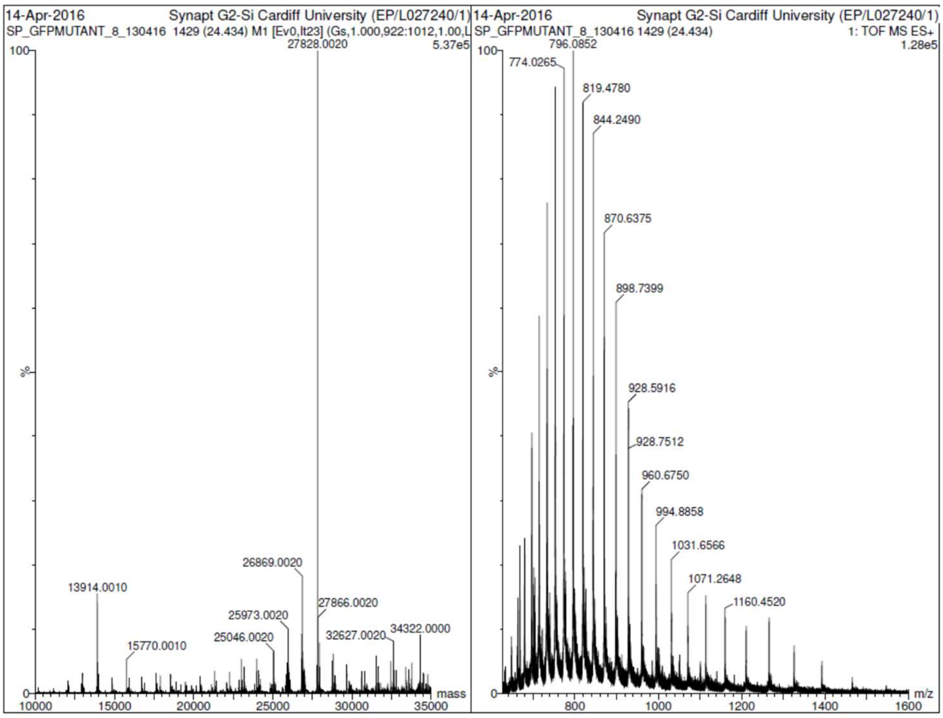

Supplementary Figure S2. LC chromatogram (a) and MS spectrum (b) of sfGFP containing an N-terminal His tag.

(a) LC chromatogram of sfGFP-TAT

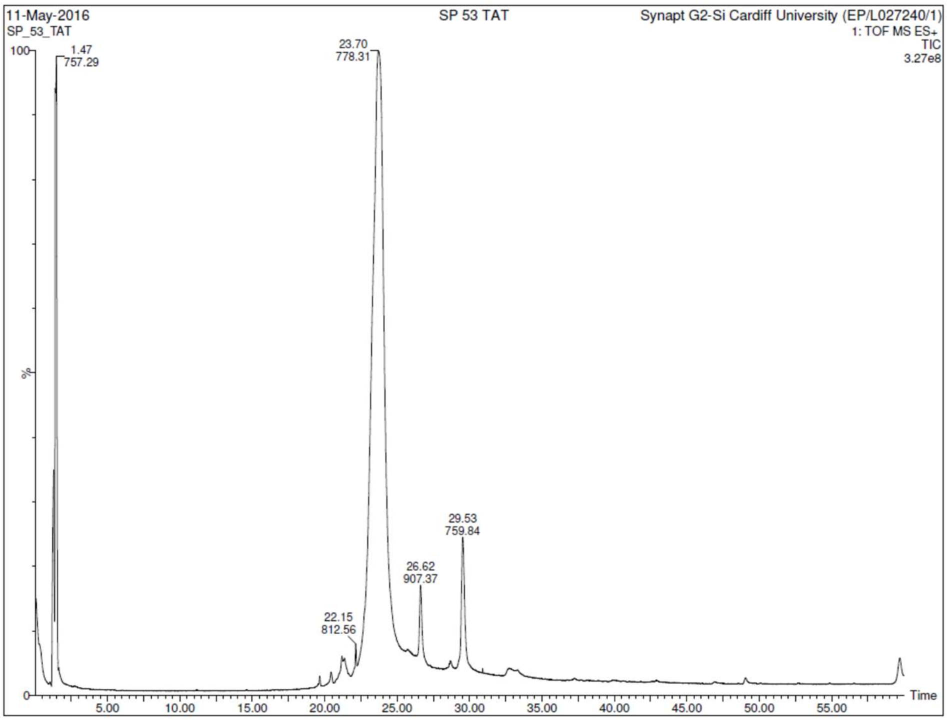

(b) MS of peak at 23.52

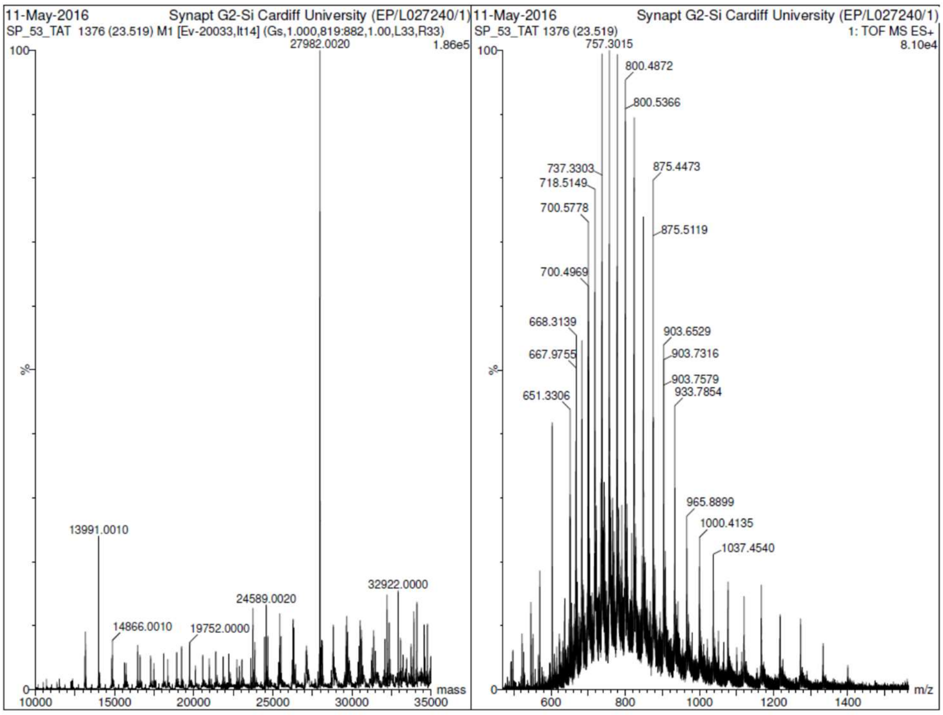

Supplementary Figure S3. LC chromatogram (a) and MS spectrum (b) of sfGFP-TAT containing an N-terminal His tag.

(a) LC chromatogram of sfGFP-Transportan

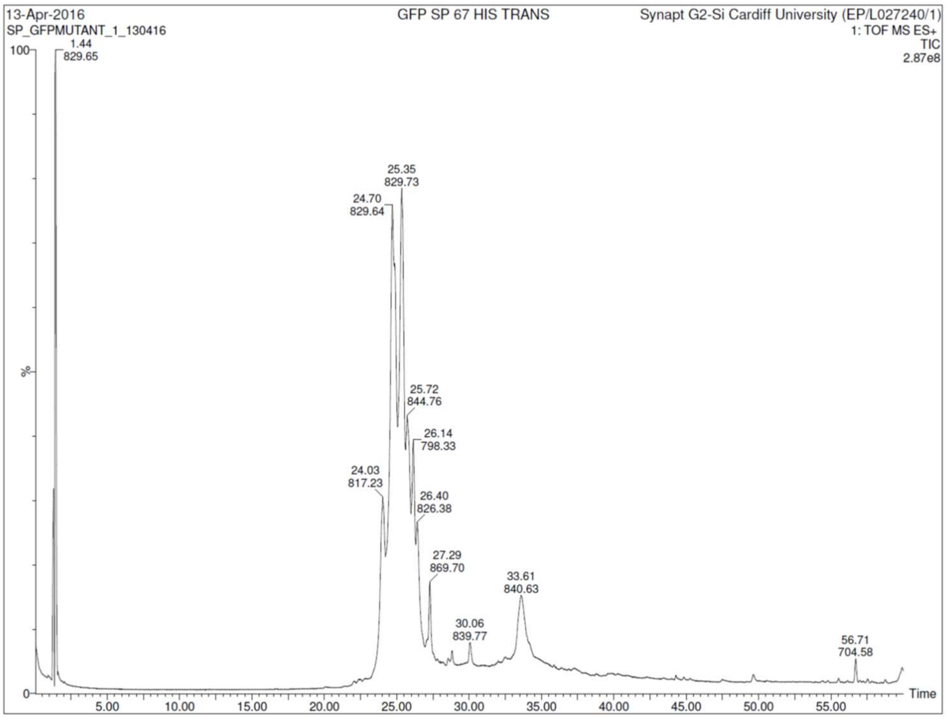

(b) MS of peak at 24.06

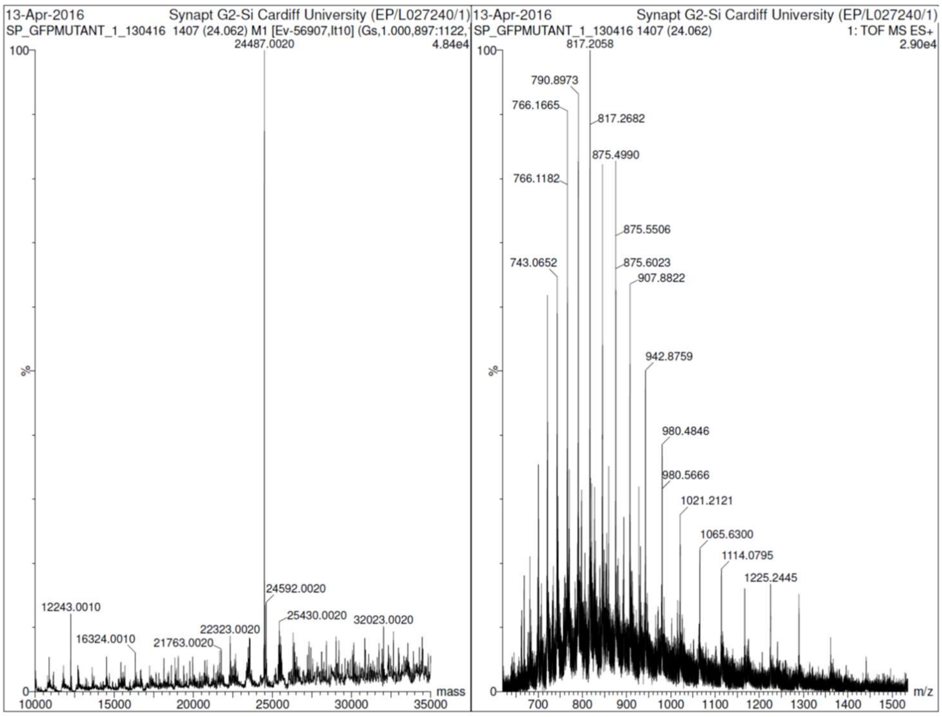

(c) MS of peak at 24.67

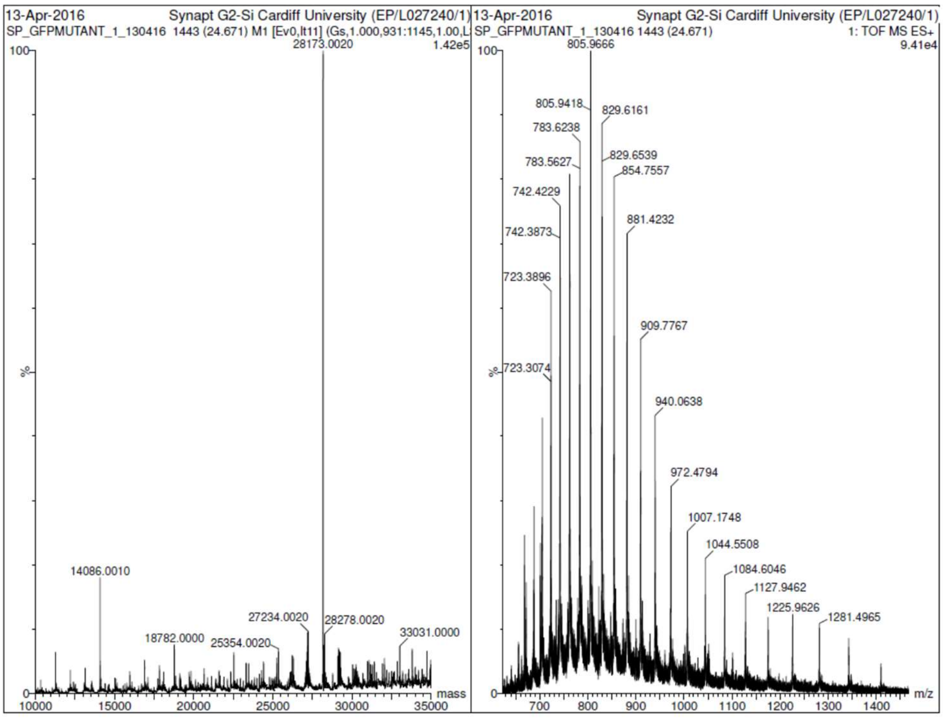

(d) MS of peak at 25.35

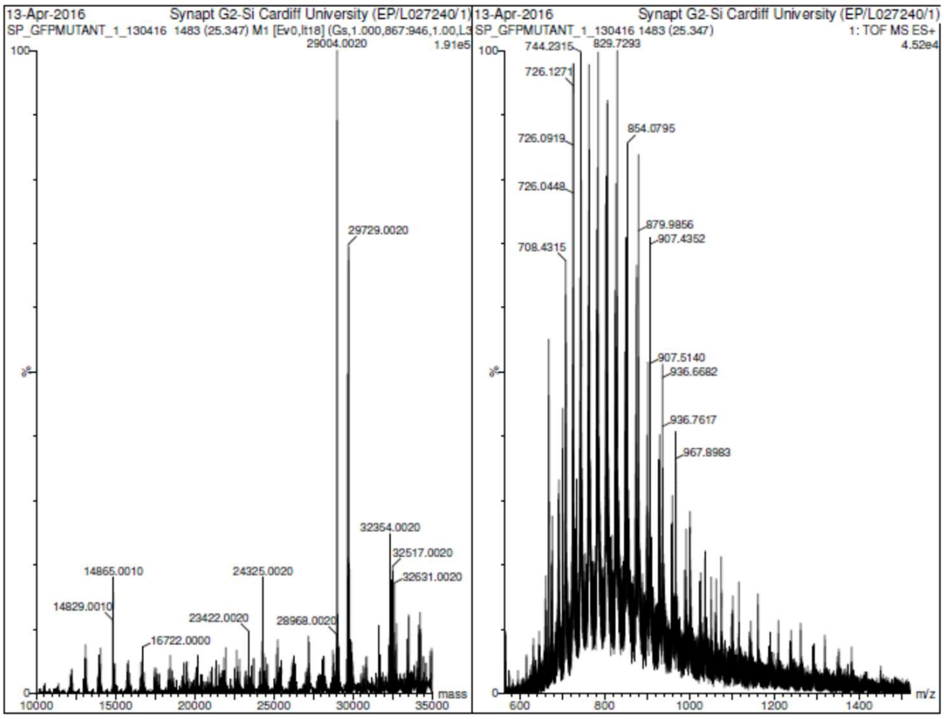

(e) MS of peak at 25.75

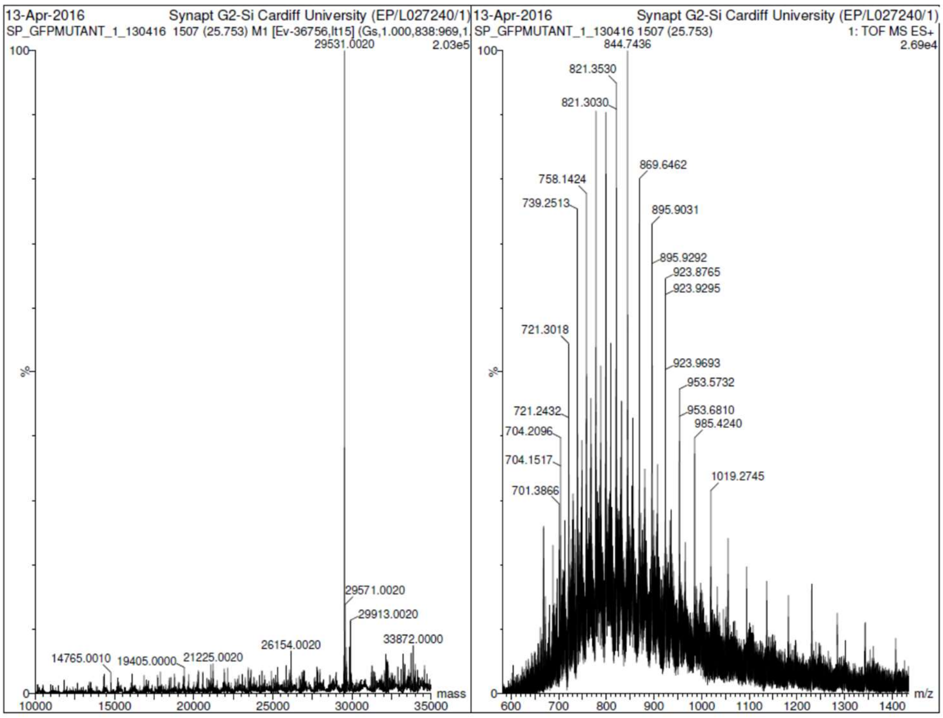

(f) MS of peak at 26.15

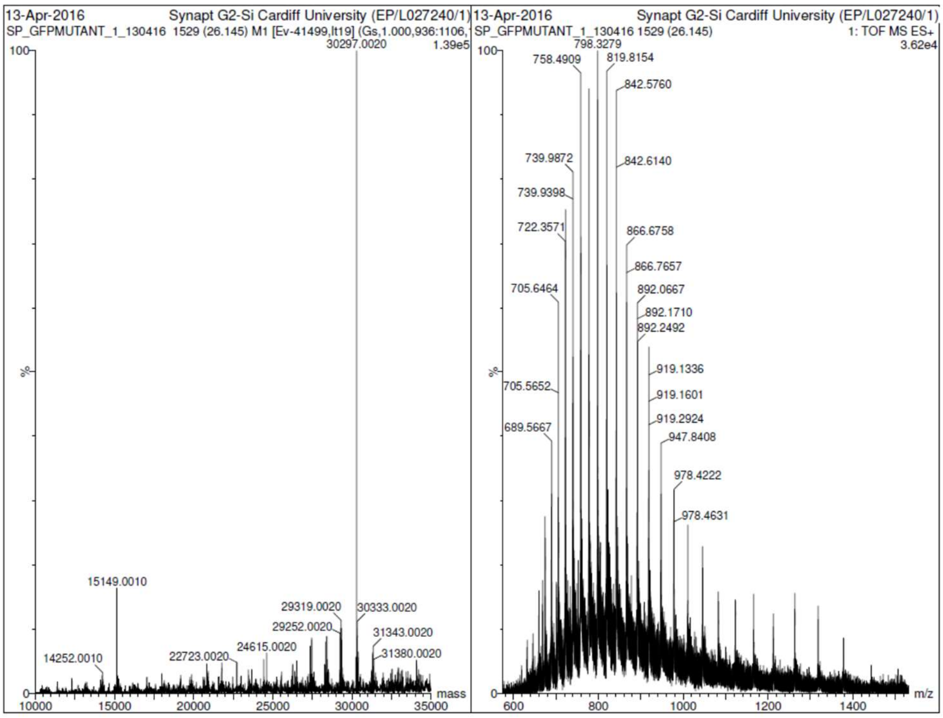

(g) MS of peak at 26.40

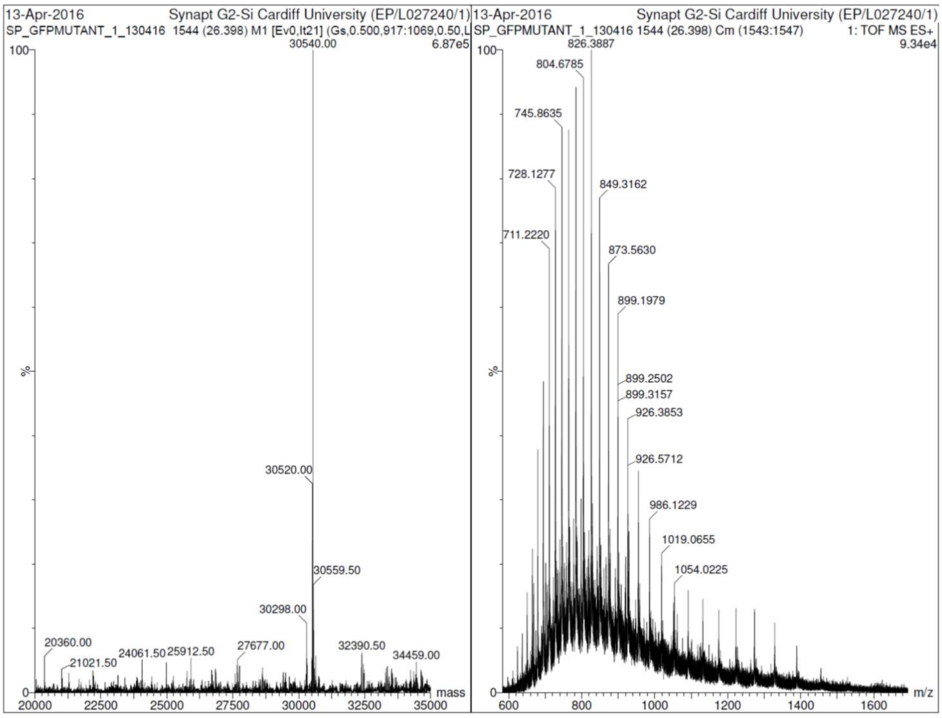

(h) MS of peak at 27.31

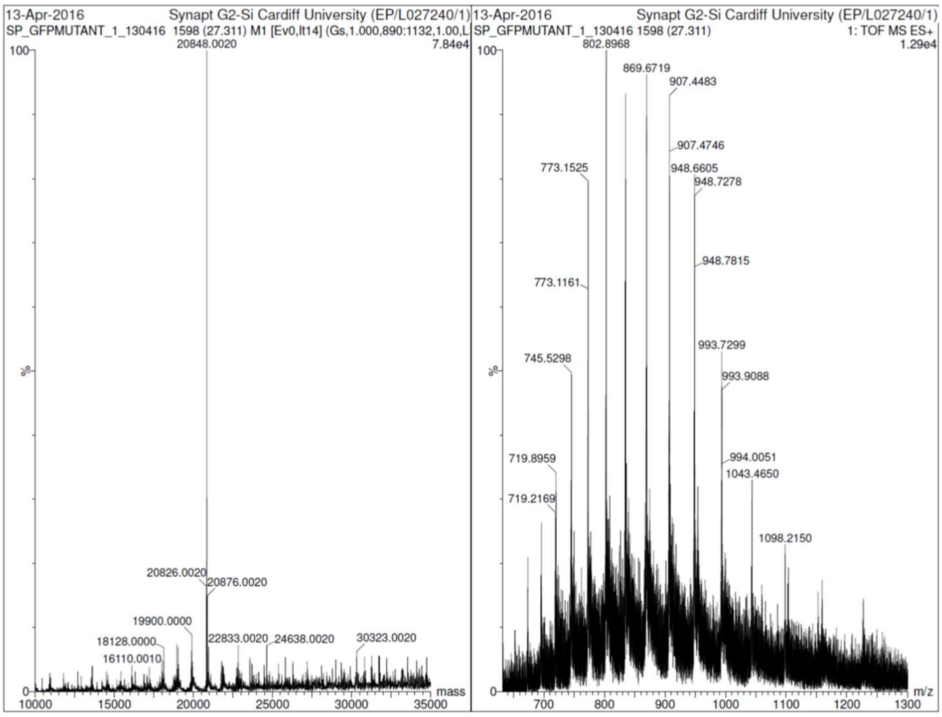

Supplementary Figure S4. LC chromatogram (a) and MS spectra (b-h) of sfGFP-Transportan containing an N-terminal His tag.

(a) LC chromatogram of sfGFP-R10

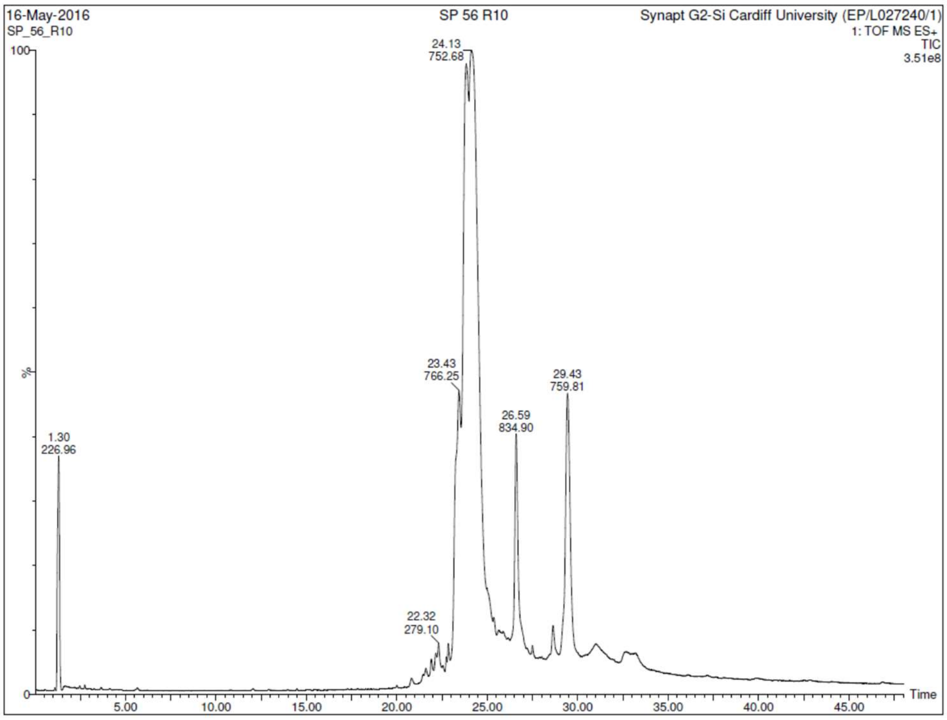

(b) MS of peak at 23.47

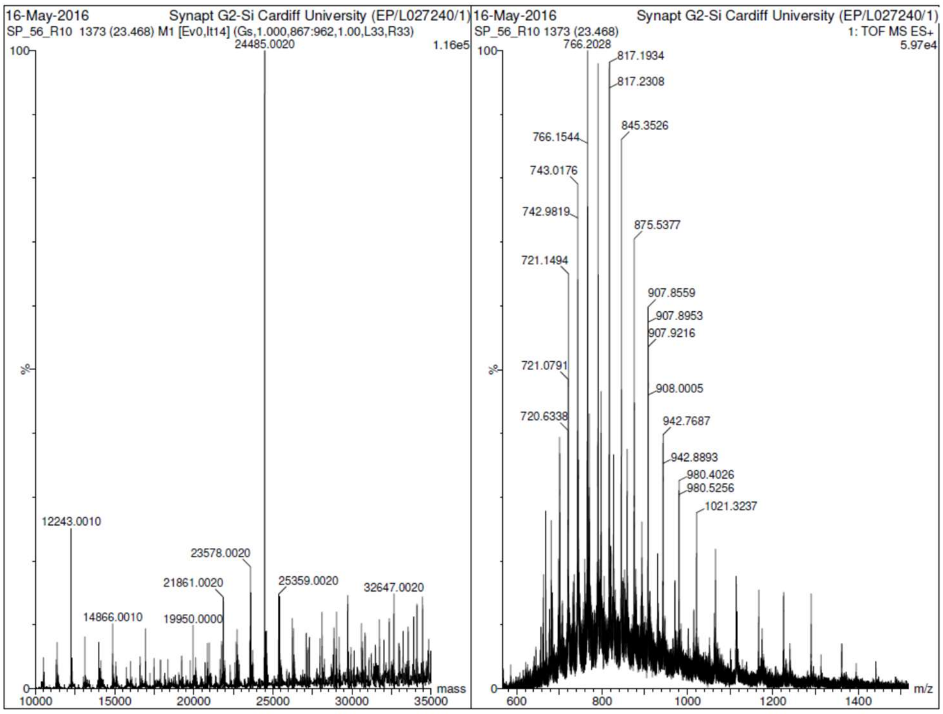

(c) MS of peak at 23.82

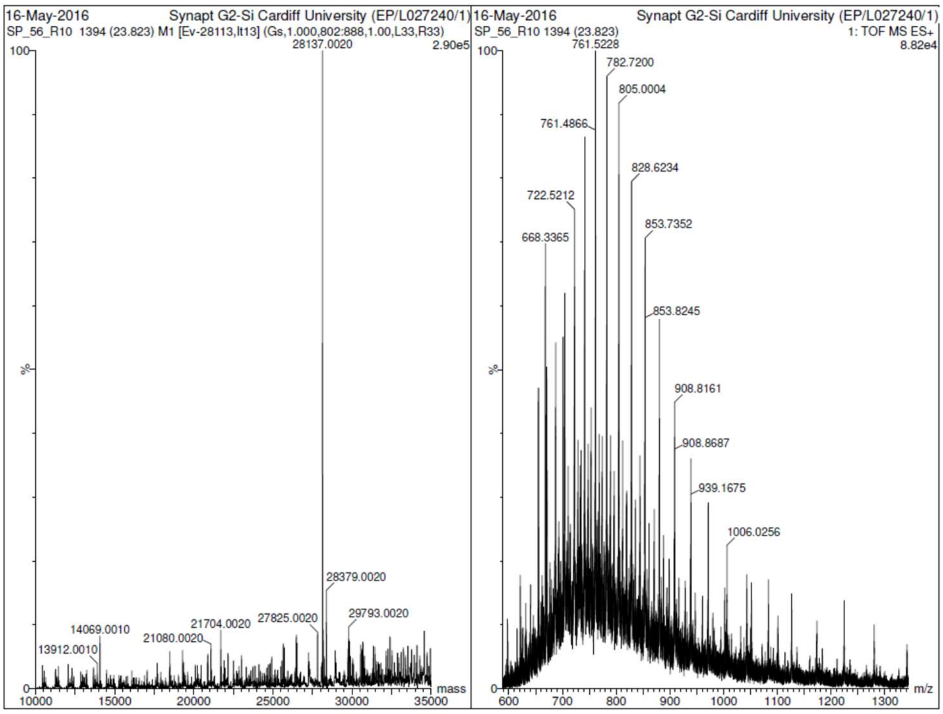

(d) MS of peak at 24.16

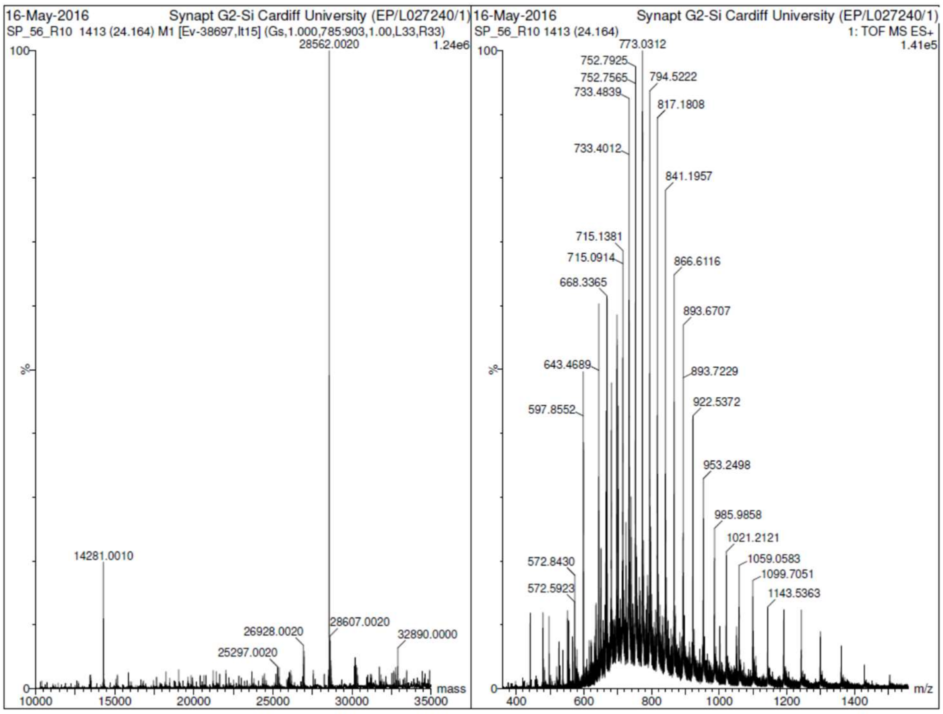

Supplementary Figure S5. LC chromatogram (a) and MS spectra (b-d) of sfGFP-R10 containing an N-terminal His tag.

(a) DNA sequence of His-eGFP-CPP

ATGCACCACCACCACCACCACATGGTGAGCAAGGGCGAGGAGCTGTTCACCGGGGTGGTGCCCATCCTGGTCGAGCT
GGACGGCGACGTAAACGGCCACAAGTTCAGCGTGTCCGGCGAGGGCGAGGGCGATGCCACCTACGGCAAGCTGACC
CTGAAGTTCATCTGCACCACCGGCAAGCTGCCCGTGCCCTGGCCCACCCTCGTGACCACCCTGACCTACGGCGTGCAG
TGCTTCAGCCGCTACCCCGACCACATGAAGCAGCACGACTTCTTCAAGTCCGCCATGCCCGAAGGCTACGTCCAGGAG
CGCACCATCTTCTTCAAGGACGACGGCAACTACAAGACCCGCGCCGAGGTGAAGTTCGAGGGCGACACCCTGGTGAA
CCGCATCGAGCTGAAGGGCATCGACTTCAAGGAGGACGGCAACATCCTGGGGCACAAGCTGGAGTACAACTACAAC
AGCCACAACGTCTATATCATGGCCGACAAGCAGAAGAACGGCATCAAGGTGAACTTCAAGATCCGCCACAACATCGA
GGACGGCAGCGTGCAGCTCGCCGACCACTACCAGCAGAACACCCCCATCGGCGACGGCCCCGTGCTGCTGCCCGACA
ACCACTACCTGAGCACCCAGTCCGCCCTGAGCAAAGACCCCAACGAGAAGCGCGATCACATGGTCCTGCTGGAGTTC
GTGACCGCCGCCGGGATCACTCTCGGCATGGACGAGCTGTACAAG[CPP sequence]TGA

**No CPP**	-
**Penetratin**	CGTCAAATTAAAATCTGGTTCCAGAACCGCCGTATGAAATGGAAGAAA
**R8**	CGTCGTCGTCGTCGTCGTCGTCGT
**TAT**	TACGGTCGTAAAAAACGTCGTCAGCGTCGTCGT
**Transportan**	GGCTGGACCCTGAACAGCGCGGGCTATCTGCTGGGCAAAATTAACCTGAAAGCGCTGGCGGCG
	CTGGCGAAAAAAATTCTG
**Xentry**	CTGTGCCTGCGCCCGGTGGGC
**cR8**	TGCCGTCGTCGTCGTCGTCGTCGTCGTTGC
**cTAT**	TGCTACGGTCGTAAAAAACGTCGTCAGCGTCGTCGTTGC
**HA-TAT**	GGTGATATTATGGGTGAATGGGGTAATGAAATCTTTGGTGCGATTGCCGGTTTTCTGGGTTACG
	GTCGTAAAAAACGTCGTCAGCGTCGTCGT

(b) Amino acid sequence of His-eGFP-CPP

MHHHHHHMVSKGEELFTGVVPILVELDGDVNGH
KFSVSGEGEGDATYGKLTLKFICTTGKLPVPWPTLV
TTLTYGVQCFSRYPDHMKQHDFFKSAMPEGYVQ
ERTIFFKDDGNYKTRAEVKFEGDTLVNRIELKGIDF
KEDGNILGHKLEYNYNSHNVYIMADKQKNGIKVN
FKIRHNIEDGSVQLADHYQQNTPIGDGPVLLPDN
HYLSTQSALSKDPNEKRDHMVLLEFVTAAGITLG
MDELYK[CPP sequence]*

**No CPP**	-
**Penetratin**	RQIKIWFQNRRMKWKK
**R8**	RRRRRRRR
**TAT**	YGRKKRRQRRR
**Transportan**	GWTLNSAGYLLGKINLKALAALAKKIL
**Xentry**	LCLRPVG
**cR8**	CRRRRRRRRC
**cTAT**	CYGRKKRRQRRRC
**HA-TAT**	GDIMGEWGNEIFGAIAGFLGYGRKKRRQRRR

Supplementary Figure S6. DNA (a) and amino acid (b) sequence of His-eGFP-CPP fusion proteins expressed from pEV vectors in *E. coli* BL21(DE3)pLysS cells.

(a) eGFP

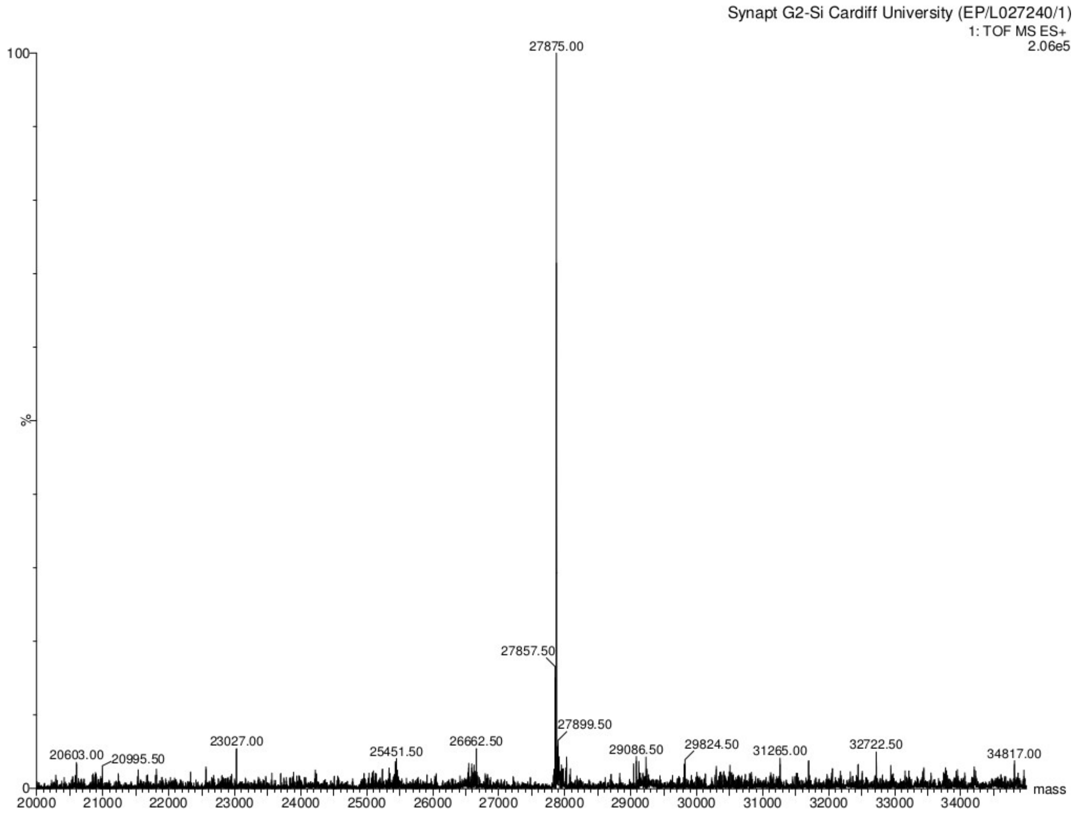

(b) eGFP-Penetratin

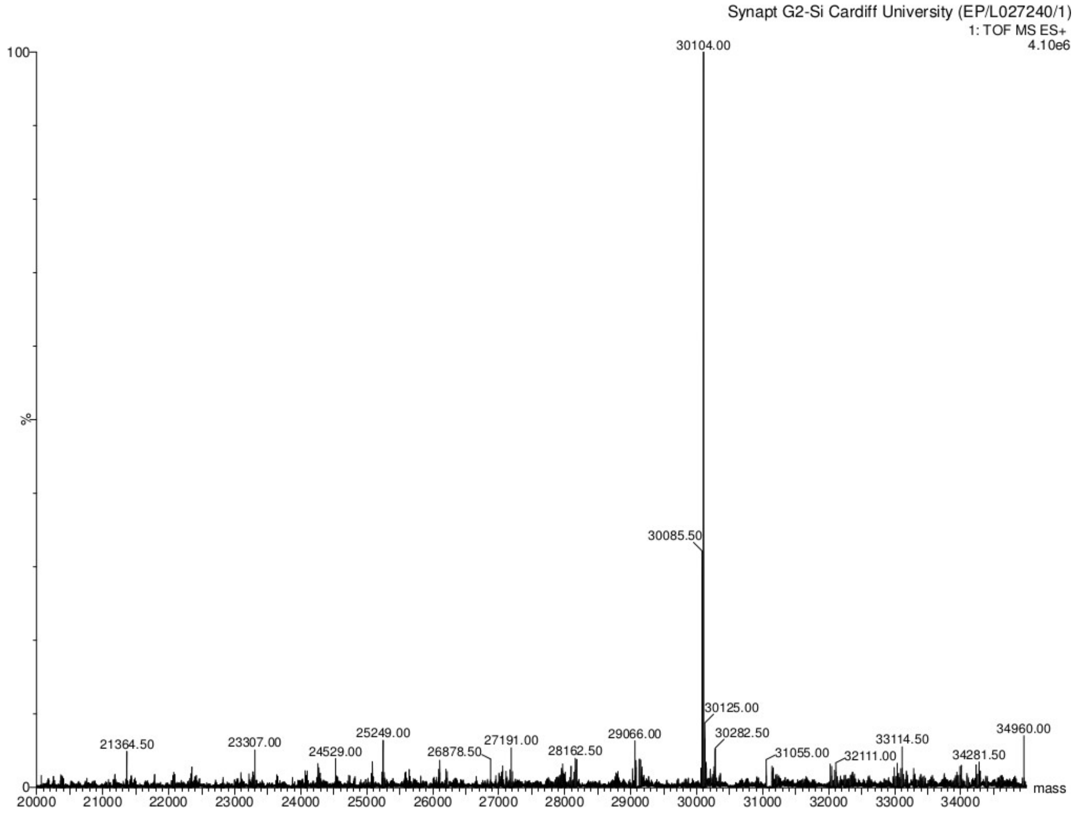

(c) R8

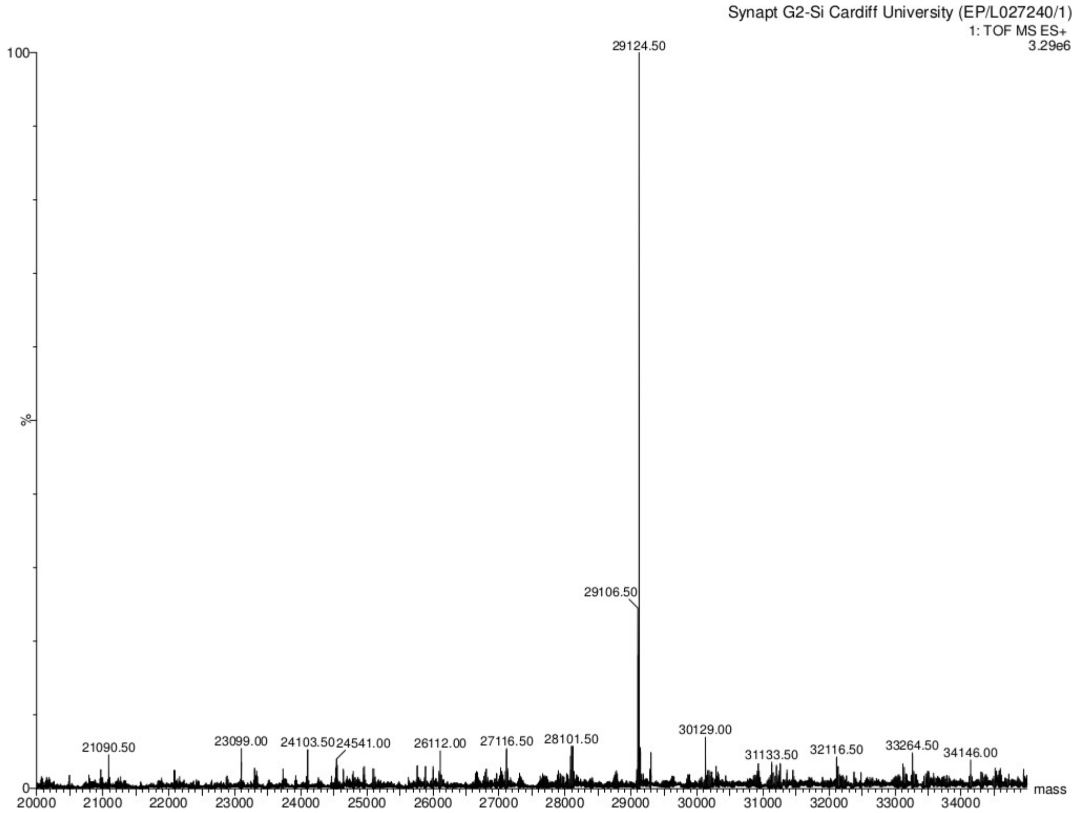

(d) eGFP-TAT

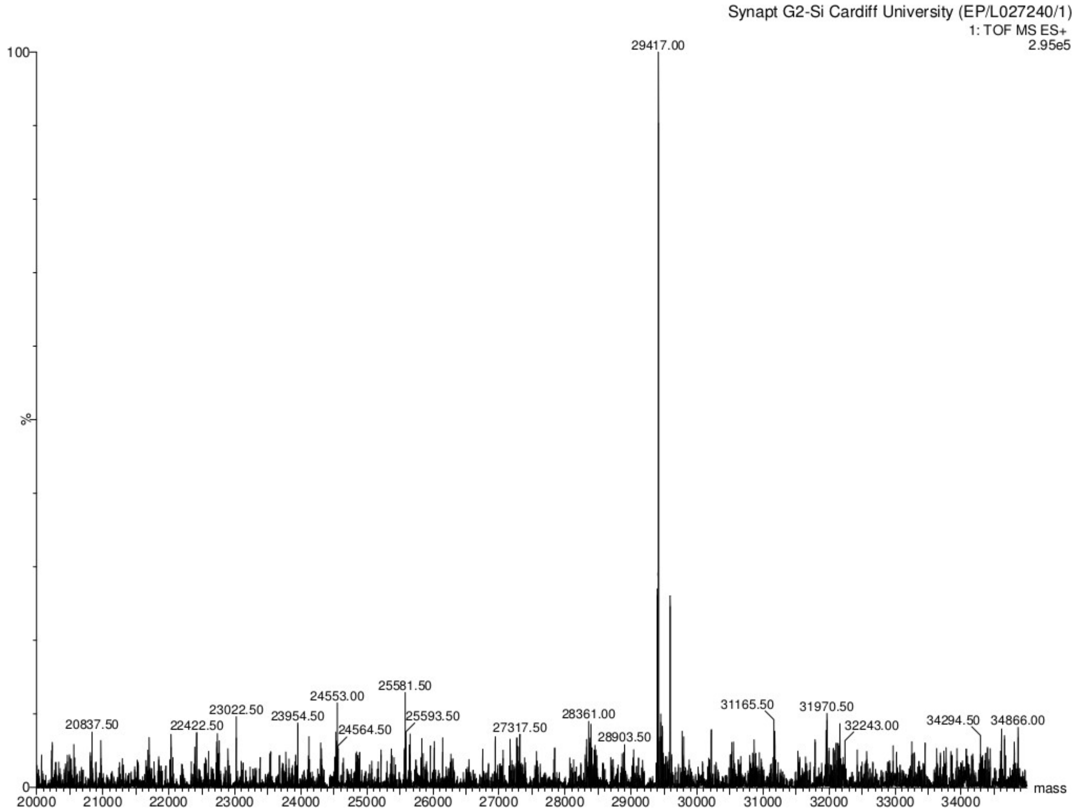

(e) eGFP-Transportan

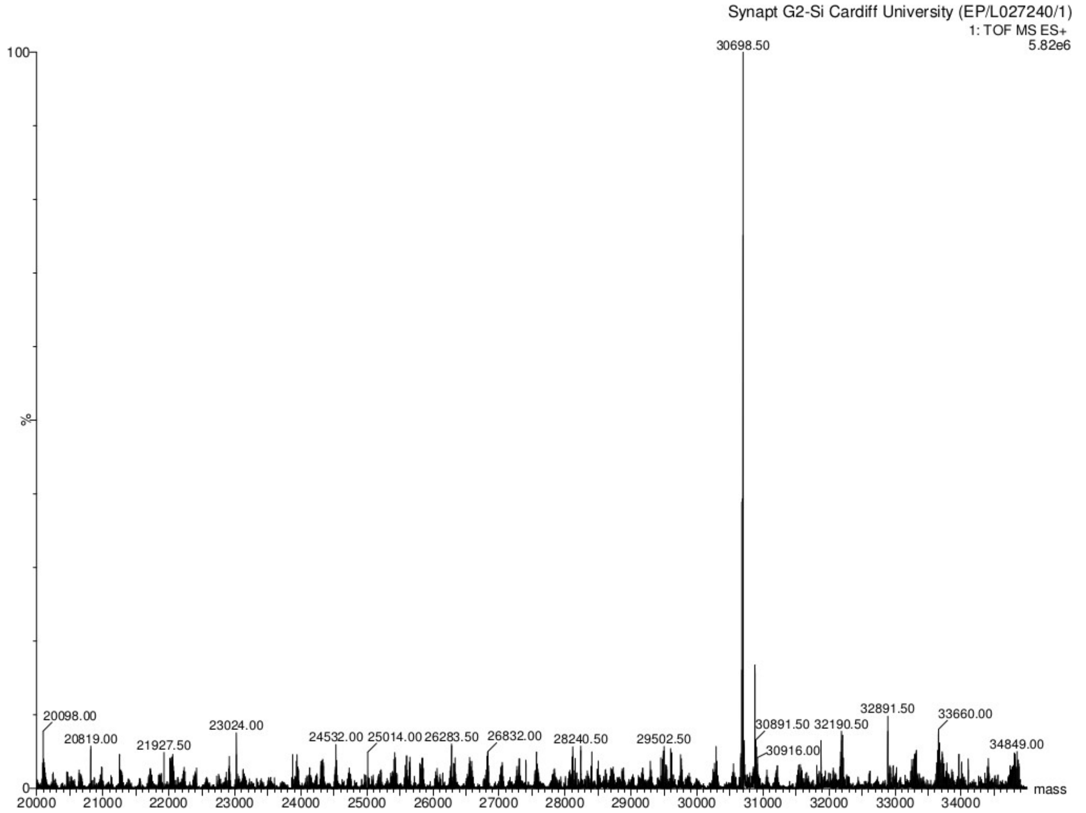

(f) eGFP-Xentry

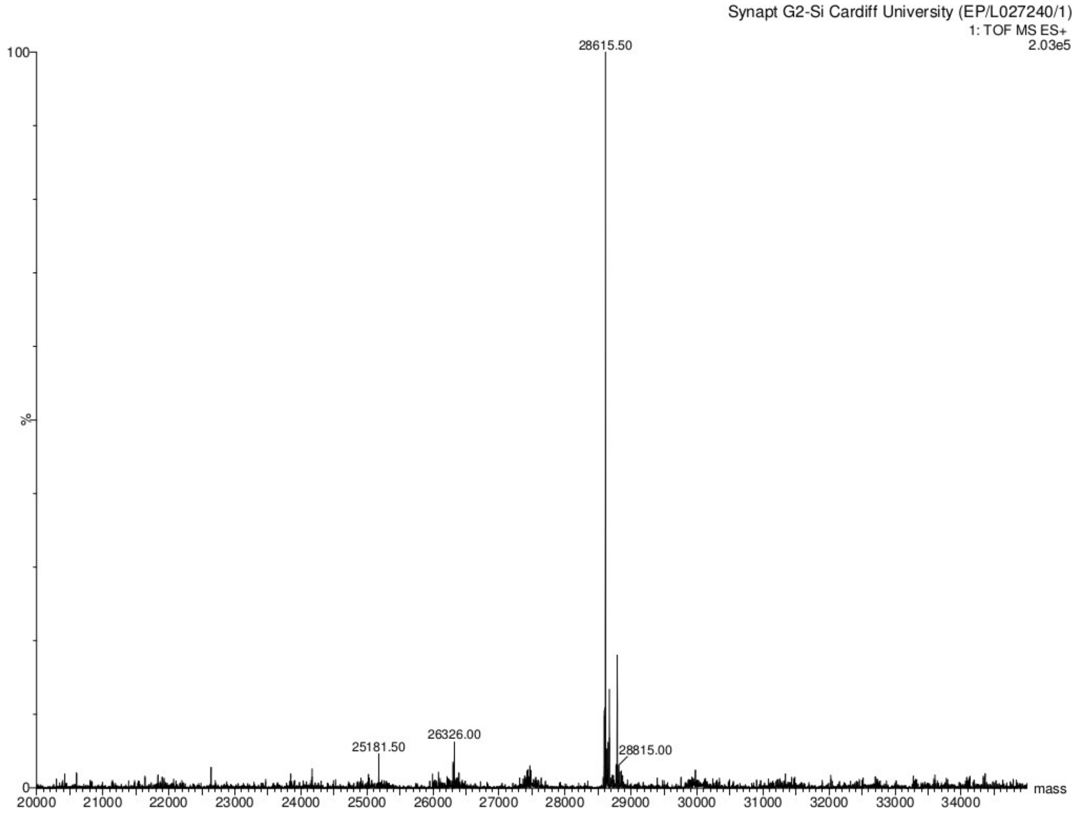

(g) eGFP-cR8

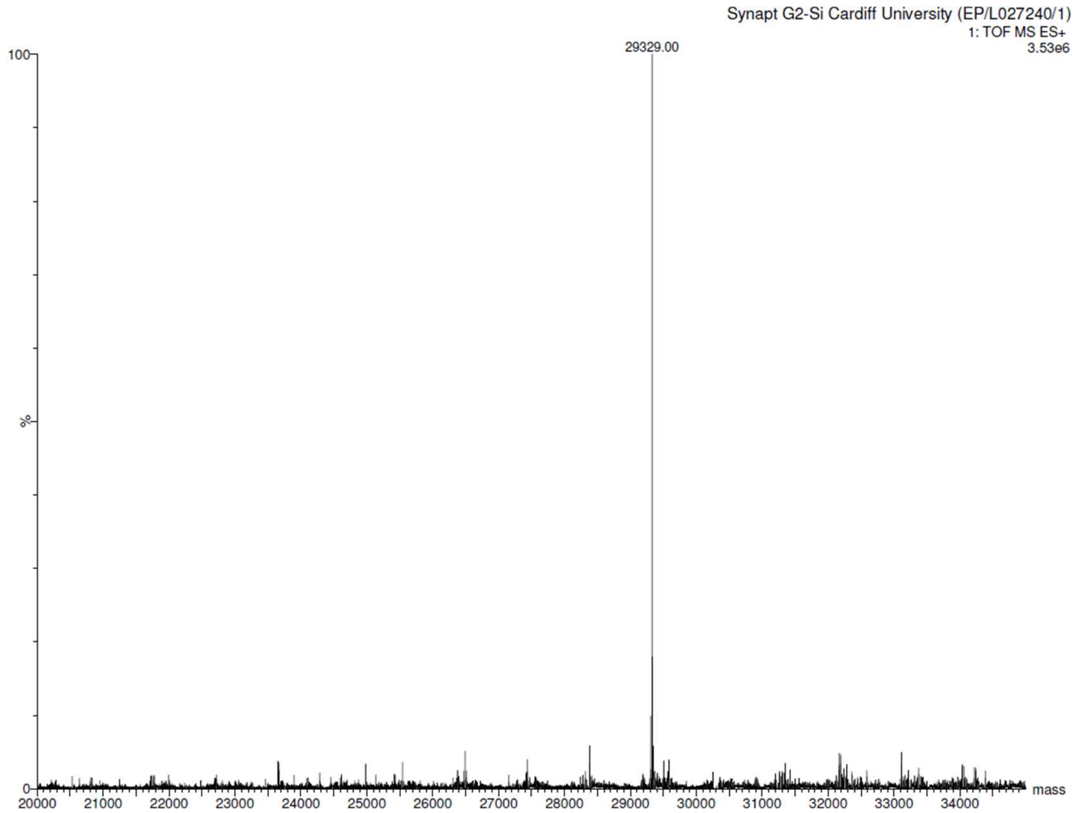

(h) eGFP-cTAT

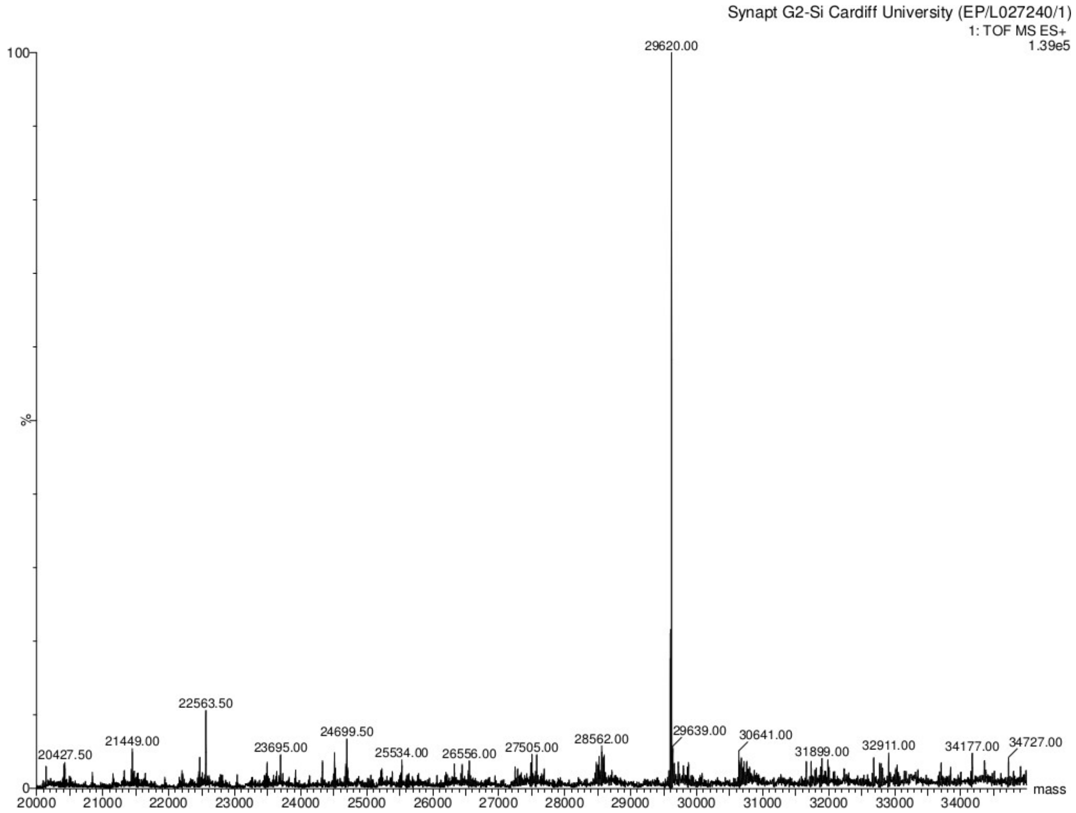

(i) eGFP-HA-TAT

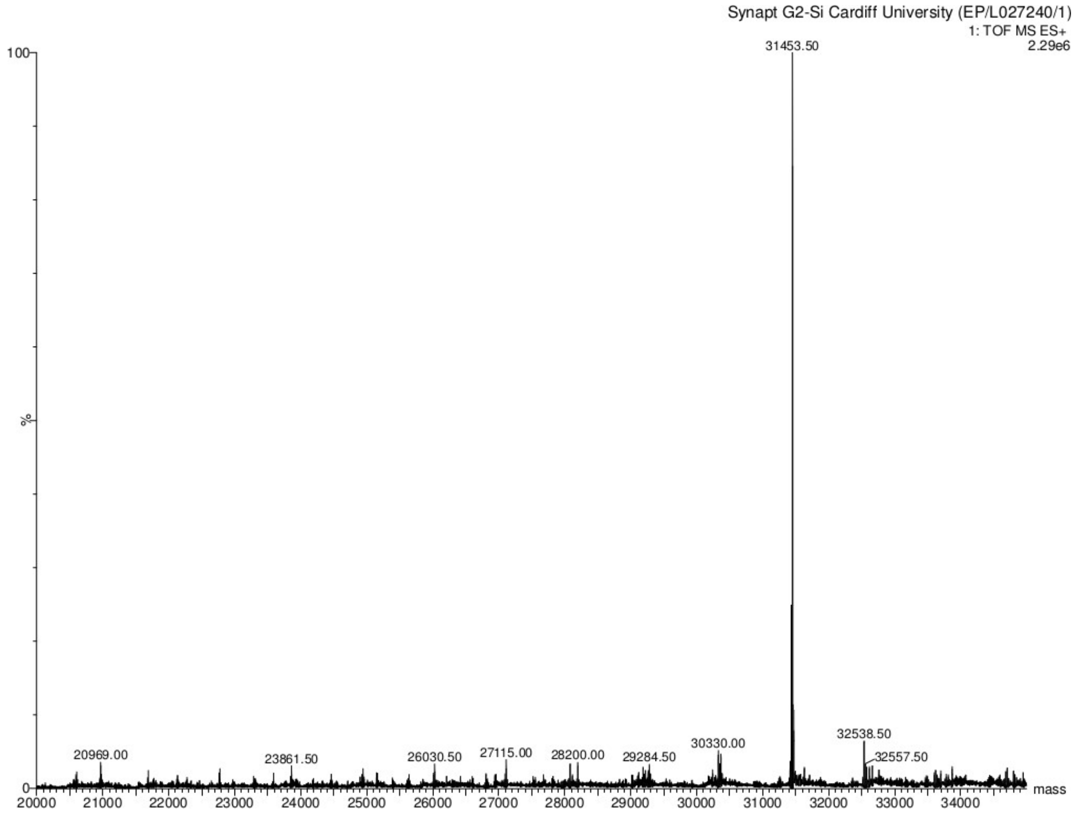

Supplementary Figure S7. MS Spectra of eGFP-CPP fusion proteins. (a) No CPP. (b) Penetratin. (c) R8. (d) TAT. (e) Transportan. (f) Xentry. (g) cR8. (h) cTAT. (i) HA-TAT.

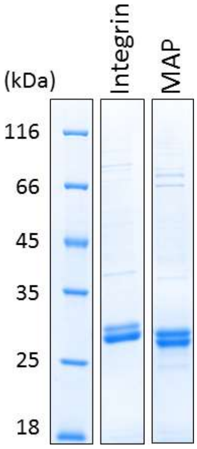

	DNA Sequence	Amino Acid Sequence
**Integrin**	AAACTGGCGCTGAAACTGGCGCTGAAAGC GCTGAAAGCGGCGCTGAAACTGGCG	VTVLALGALAGVGVG
**MAP**	GTGACCGTGCTGGCGCTGGGCGCGCTGGC GGGCGTGGGCGTGGGC	KLALKLALKALKAALKLA

Supplementary Figure S8. SDS-PAGE of eGFP-MAP and eGFP-Integrin after purification by Ni column.

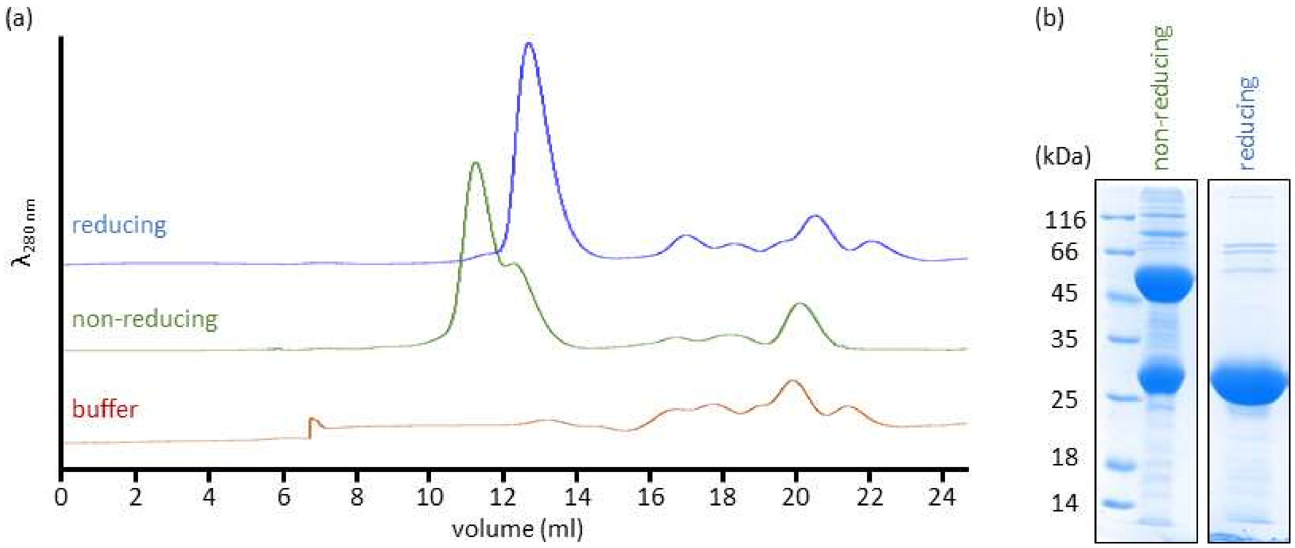

Supplementary Figure S9. eGFP-Xentry exists as dimer through a disulphide linkage from the cysteine residue in the Xentry sequence. (a) Analytical size-exclusion chromatography and (b) non-reducing SDS-PAGE of eGFP-Xentry under non-reducing and reducing conditions. Size-exclusion chromatography was performed on an NGC Chromatography System (Bio-Rad). Superdex 75 10/300 GL (24 mL, GE Healthcare) was equilibrated with PBS. Sample was then loaded on the column, followed by elution with PBS at a flow rate of 0.5 mL/min. To reduce the disulphide bond, the sample was treated with β-mercaptoethanol (10 µM) at 20°C for 1 h.

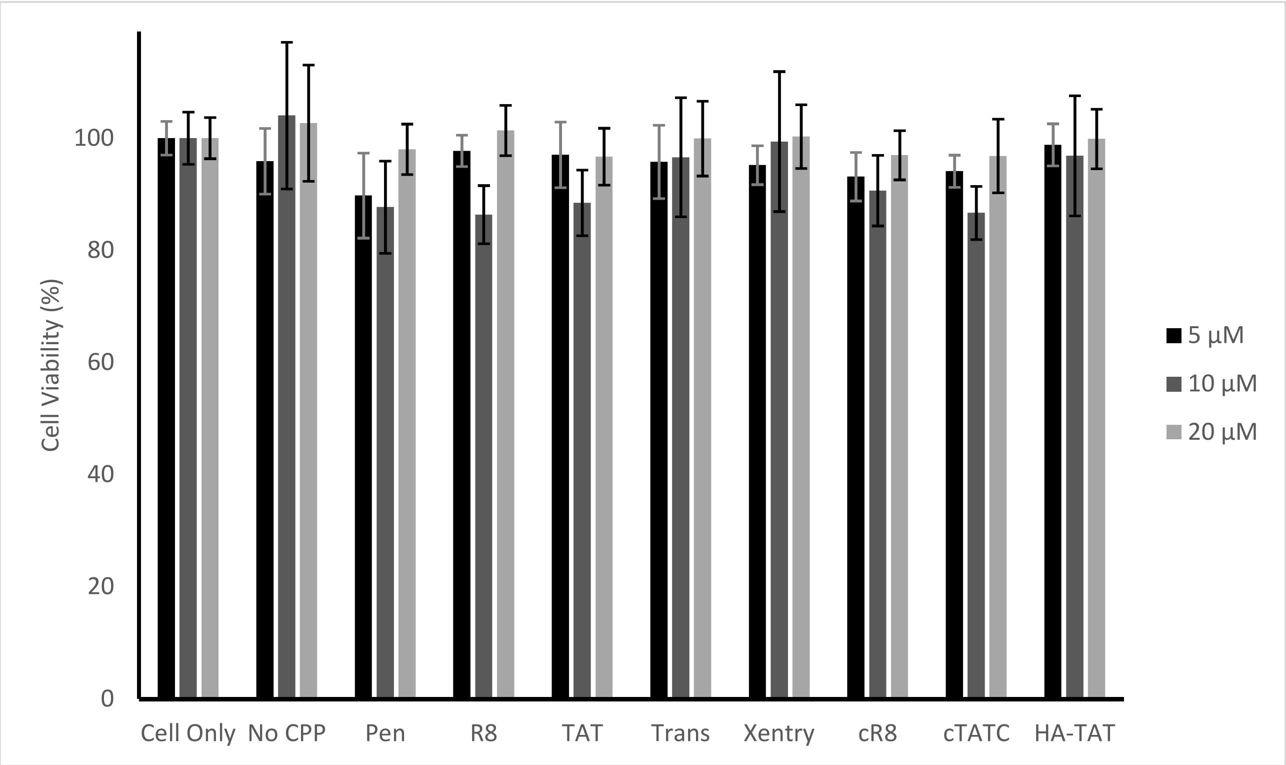

Supplementary Figure S10. Viability of HeLa cells treated with 5 µM, 10 µM and 20 µM of eGFP-CPP at 37 °C for 24 h, as monitored using CellTiter-Blue (Promega, G8080) following the manufacturer's protocol.

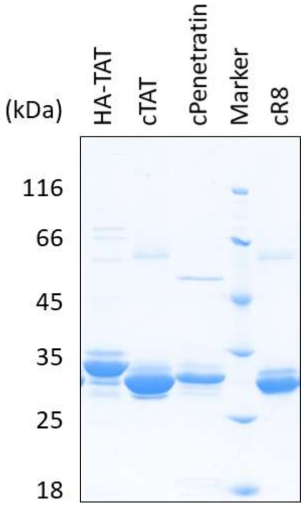

Supplementary Figure S11. SDS-PAGE of eGFP proteins fused to a modified CPP.

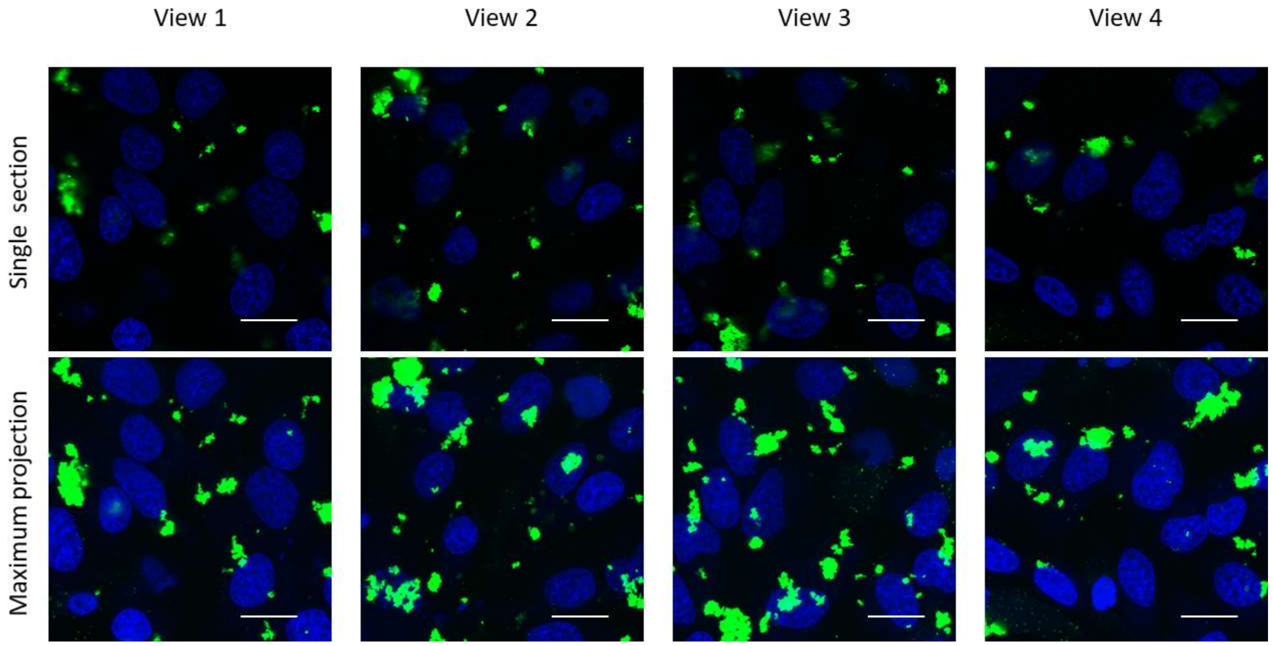

Supplementary Figure S12. Confocal microscopy images of HeLa cells treated with 10 μM eGFP-cPenetratin at 37 °C for 1 h before imaging. Nucleus were stained with Hoechst which shows blue fluorescence. Scale bars represent 20 μm.

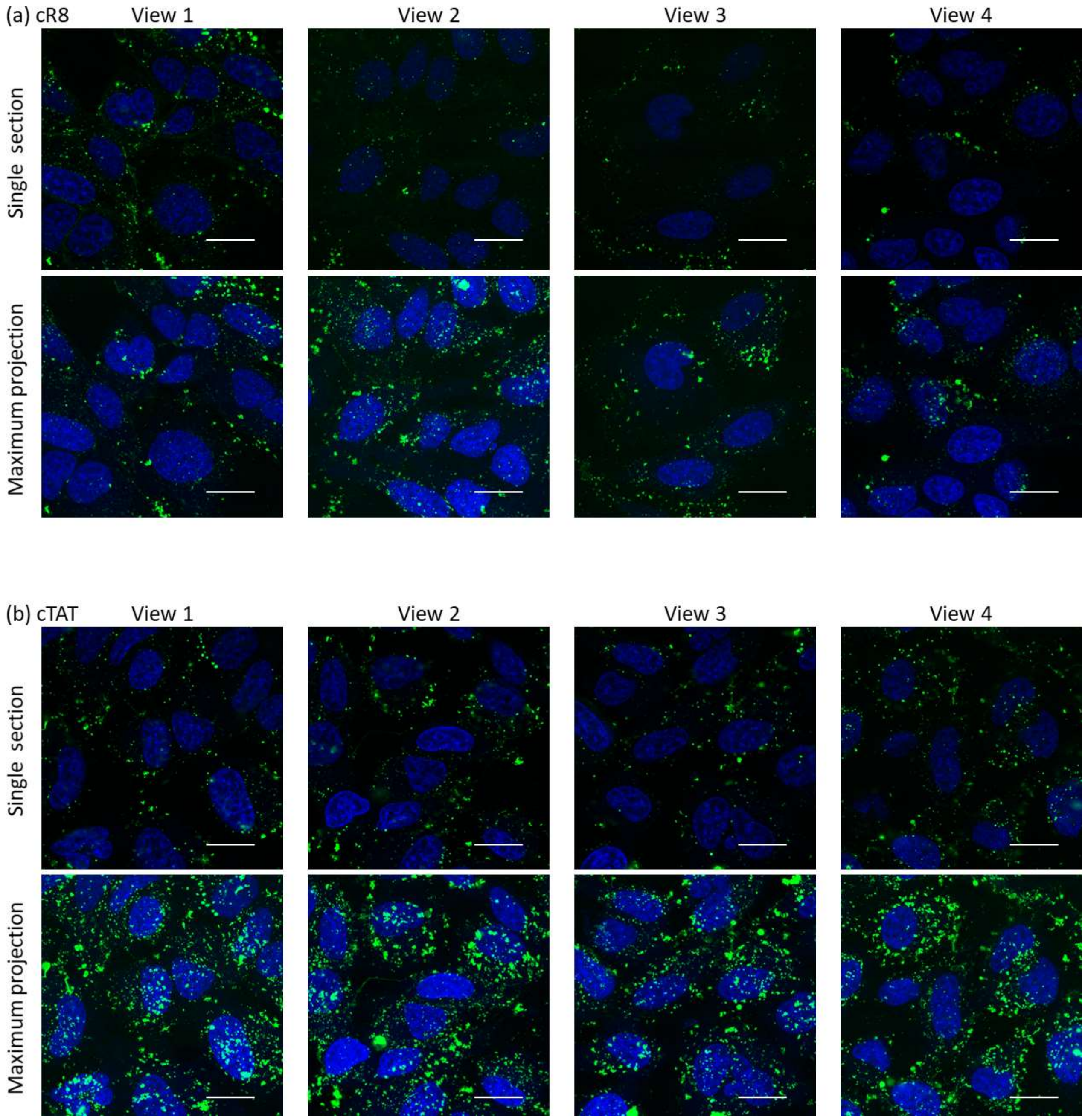

Supplementary Figure S13. Confocal microscopy images of HeLa cells treated with 100 μM eGFP-cR8 (a) or eGFP-cTAT (b) at 37 °C for 1 h before imaging. Nucleus were stained with Hoechst which shows blue fluorescence. Gain of the green fluorescence is reduced in comparison to other images in order to observe the fluorescence pattern. Scale bars represent 20 μm.

Supplementary Table S1. Fluorescence intensity of cells treated with eGFP-CPP fusion proteins[a]

	HeLa	HEK	10T1/2	HepG2
Cell Only[b]	6 ± 0	5 ± 1	15 ± 3	5 ± 1
No CPP	14 ± 4	9 ± 0	31 ± 1	9 ± 1
Penetratin	55 ± 5	40 ± 2	237 ± 40	74 ± 19
R8	95 ± 28	72 ± 4	358 ± 69	116 ± 17
TAT	51 ± 3	45 ± 1	235 ± 35	76 ± 14
Transportan	89 ± 12	93 ± 17	171 ± 6	959 ± 227
Xentry	27 ± 2	16 ± 1	57 ± 10	43 ± 19

[a] Cells were treated with 10 μM of eGFP-CPP fusion proteins at 37 °C for 1 h before analysis by flow cytometry. Cells containing eGFP-CPP fusion proteins showed fluorescence at 526 nm when excited with a laser of 488 nm. Geometric mean values of green fluorescence from living cells were recorded for each sample. Values shown here are mean ± standard deviation from measurements of three biological replica. [b] Cell autofluorescence values.

Supplementary Table S2. The *p*-values of two-sample t-tests to determine whether different CPP sequences have statistically significant effects in fusion-protein uptake by the same cell line. Values were calculated using the identical data as in Supplementary Table S1.[a]

**HeLa**	No CPP	Penetratin	R8	TAT	Transportan	Xentry
Cell Only	0.017416	0.000114	0.005154	0.000011	0.000282	0.000073
No CPP		0.000461	0.007318	0.000160	0.000496	0.006171
Penetratin			0.055966	0.225312	0.010046	0.001228
R8				0.044070	0.391577	0.012521
TAT					0.005663	0.000275
Transportan						0.000895

**HEK**	No CPP	Penetratin	R8	TAT	Transportan	Xentry
Cell Only	0.002742	0.000014	< 0.00001	< 0.00001	0.001046	0.000349
No CPP		0.000017	< 0.00001	< 0.00001	0.001262	0.001145
Penetratin			0.000227	0.031356	0.006747	0.000081
R8				0.000310	0.085922	0.000018
TAT					0.008974	0.000015
Transportan						0.001753

**10T1/2**	No CPP	Penetratin	R8	TAT	Transportan	Xentry
Cell Only	0.000541	0.000700	0.001058	0.000427	< 0.00001	0.002221
No CPP		0.000928	0.001268	0.000569	< 0.00001	0.011339
Penetratin			0.048747	0.476230	0.040254	0.001682
R8				0.042974	0.009229	0.001769
TAT					0.030835	0.001083
Transportan						0.000069

**HepG2**	No CPP	Penetratin	R8	TAT	Transportan	Xentry
Cell Only	0.007146	0.003749	0.000375	0.001143	0.002016	0.026127
No CPP		0.004699	0.000438	0.001447	0.002048	0.036181
Penetratin			0.041375	0.452911	0.002681	0.092394
R8				0.032357	0.003180	0.007989
TAT					0.002689	0.062107
Transportan						0.002365

[a] Results showing no statistical significance ($p > 0.05$) are highlighted.

Supplementary Table S3. The *p*-values of two-sample t-tests to determine whether different cell lines have statistically significant effects in uptake of the same fusion-protein. Values were calculated using the identical data as in Supplementary Table S1.[a]

**Penetratin**	HEK	10T1/2	HepG2
HeLa	0.012357	0.001503	0.127408
HEK		0.001098	0.036519
10T1/2			0.003207

**Transportan**	HEK	10T1/2	HepG2
HeLa	0.402711	0.000433	0.002829
HEK		0.001919	0.002892
10T1/2			0.004012

**R8**	HEK	10T1/2	HepG2
HeLa	0.150757	0.003674	0.21071
HEK		0.002074	0.011478
10T1/2			0.004161

**Xentry**	HEK	10T1/2	HepG2
HeLa	0.001832	0.006819	0.159262
HEK		0.002253	0.064862
10T1/2			0.207597

**TAT**	HEK	10T1/2	HepG2
HeLa	.021274	.000846	.038258
HEK		.000737	.01951
10T1/2			.001939

[a] Results showing no statistical significance ($p > 0.05$) are highlighted.

Supplementary Table S4. Green fluorescence intensity of cells treated with eGFP proteins fused to a modified CPP[a]

	**HeLa**	**HEK**	**10T1/2**	**HepG2**
Cell Only	3 ± 0	2 ± 0	4 ± 1	2 ± 0
No CPP	6 ± 0	9 ± 3	13 ± 1	12 ± 3
R8	73 ± 8	49 ± 5	381 ± 26	117 ± 8
cR8	293 ± 60	114 ± 13	956 ± 211	247 ± 19
TAT	61 ± 4	41 ± 2	325 ± 56	76 ± 2
cTAT	361 ± 46	152 ± 13	1005 ± 94	298 ± 34
TAT-HA	121 ± 14	98 ± 3	481 ± 64	294 ± 10

[a] The same experimental conditions as in the Supplementary Table S1.

Supplementary Table S5. The *p*-values of two-sample t-tests to determine whether different CPP modifications have statistically significant effects in fusion-protein uptake by the same cell line. Values were calculated using the identical data as in Supplementary Table S4.[a]

**HeLa**	No CPP	R8	cR8	TAT	cTAT	TAT-HA
Cell Only	0.000294	0.000123	0.001220	0.000020	0.000190	0.000127
No CPP		0.000149	0.001274	0.000026	0.000198	0.000142
R8			0.003466	0.071197	0.000469	0.006473
cR8				0.002816	0.136549	0.008484
TAT					0.000386	0.002068
cTAT						0.001034

**HEK**	No CPP	R8	cR8	TAT	cTAT	TAT-HA
Cell Only	0.005756	0.000017	< 0.00001	0.000489	< 0.00001	< 0.00001
No CPP		0.000095	0.000012	0.001465	< 0.00001	< 0.00001
R8			0.000226	0.386961	0.000048	0.000033
cR8				0.000872	0.016392	0.086019
TAT					0.000137	0.000695
cTAT						0.001677

**10T1/2**	No CPP	R8	cR8	TAT	cTAT	TAT-HA
Cell Only	0.000618	0.000017	0.001557	0.000621	0.000056	0.000229
No CPP		0.000018	0.001611	0.000689	0.000058	0.000246
R8			0.009379	0.137017	0.000407	0.053765
cR8				0.007557	0.388379	0.019241
TAT					0.000458	0.030092
cTAT						0.001424

**HepG2**	No CPP	R8	cR8	TAT	cTAT	TAT-HA
Cell Only	0.016438	< 0.00001	0.000027	< 0.00001	0.000128	0.000088
No CPP		0.000021	0.000034	0.000032	0.000149	0.000104
R8			0.000284	0.001233	0.000735	0.000640
cR8				0.000114	0.070714	0.181274
TAT					0.000392	0.000308
cTAT						0.228482

[a] Results showing no statistical significance ($p > 0.05$) are highlighted.

Supplementary Table S6. The *p*-values of two-sample t-tests to analyse if different cell lines have statistically significant effects on uptake of the same fusion-protein using the identical data as in Supplementary Table S4.[a]

**R8**	HEK	10T1/2	HepG2
HeLa	0.005077	0.000044	0.003967
HEK		< 0.00001	0.000064
10T1/2			0.000064

**cTAT**	HEK	10T1/2	HepG2
HeLa	0.001741	0.000472	0.097348
HEK		0.000109	0.002471
10T1/2			0.000278

**cR8**	HEK	10T1/2	HepG2
HeLa	0.007439	0.006489	0.183552
HEK		0.002459	0.000599
10T1/2			0.004577

**TAT-HA**	HEK	10T1/2	HepG2
HeLa	0.042747	0.000729	0.001203
HEK		0.000535	0.000493
10T1/2			0.006675

**TAT**	HEK	10T1/2	HepG2
HeLa	0.002919	0.001313	0.010509
HEK		0.000237	0.018260
10T1/2			0.001602

[a] Results showing no statistical significance ($p > 0.05$) are highlighted.
